# Supplementary material for: Therapy of genomic unstable solid tumours (WHO grade 3/4）in clinical stage III/IV using individualised neoantigen tumour peptides-INP trial (individualised neoantigen tumour peptides immunotherapy): study protocol for an open-label, non-randomised, prospective, single-arm trial
Source: BMJ Open. 2022 Jun 10;12(6):e055742. doi: 10.1136/bmjopen-2021-055742 (PMC9189849; doi:10.1136/bmjopen-2021-055742)
Supplement: Author's manuscript [file bmjopen-2021-055742.draft_revisions.pdf]

BMJ Open is committed to open peer review. As part of this commitment we make the peer review history of every article we publish publicly available.

When an article is published we post the peer reviewers' comments and the authors' responses online. We also post the versions of the paper that were used during peer review. These are the versions that the peer review comments apply to.

The versions of the paper that follow are the versions that were submitted during the peer review process. They are not the versions of record or the final published versions. They should not be cited or distributed as the published version of this manuscript.

BMJ Open is an open access journal and the full, final, typeset and author-corrected version of record of the manuscript is available on our site with no access controls, subscription charges or pay-per-view fees (http://bmjopen.bmj.com).

If you have any questions on BMJ Open's open peer review process please email info.bmjopen@bmj.com

BMJ Open

Therapy of genomic unstable solid tumors（WHO grade 3/4）in clinical stage III/IV using individualized neoantigen tumor peptides - INP Trial (Individualized neoantigen tumor peptides Immunotherapy):study protocol for an open-label, non- randomised, prospective, single-arm trial

Journal:	*BMJ Open*
Manuscript ID	bmjopen-2021-055742
Article Type:	Protocol
Date Submitted by the Author:	24-Jul-2021

Complete List of Authors:	Wang, Ling; Chongqing Key Laboratory of Translational Research for Cancer Metastasis and Individualized Treatment, Chongqing University Cancer Hospital,, Phase I Clinical Trial Ward<br>Tang, jiaxi; Chongqing Key Laboratory of Translational Research for Cancer Metastasis and Individualized Treatment, Chongqing University Cancer Hospital, Anesthesiology<br>Chen, Xia; Chongqing Key Laboratory of Translational Research for Cancer Metastasis and Individualized Treatment, Chongqing University Cancer Hospital, Department of Phase I Clinical Trial Ward<br>Zhao, Juan; Chongqing Key Laboratory of Translational Research for Cancer Metastasis and Individualized Treatment, Chongqing University Cancer Hospital, Department of Phase I Clinical Trial Ward<br>Tang, Wanyan; Chongqing Key Laboratory of Translational Research for Cancer Metastasis and Individualized Treatment, Chongqing University Cancer Hospital, Department of Phase I Clinical Trial Ward<br>Liao, Bin; Chongqing Key Laboratory of Translational Research for Cancer Metastasis and Individualized Treatment, Chongqing University Cancer Hospital, Department of Phase I Clinical Trial Ward<br>Nian, Weiqi; Chongqing Key Laboratory of Translational Research for Cancer Metastasis and Individualized Treatment, Chongqing University Cancer Hospital, Department of Phase I Clinical Trial Ward

Keywords:	ONCOLOGY, IMMUNOLOGY, Protocols & guidelines < HEALTH SERVICES ADMINISTRATION & MANAGEMENT

SCHOLARONE™
Manuscripts

Therapy of genomic unstable solid tumors（WHO grade 3/4）in clinical stage III/IV using individualized neoantigen tumor peptides - INP Trial (Individualized neoantigen tumor peptides Immunotherapy):study protocol for an open-label, non-randomised, prospective, single-arm trial

Ling Wang[1 †] jiaxi Tang[2 †], Xia Chen[1],Juan Zhao[1], Wanyan Tang[1], Bin Liao[1], Weiqi Nian[1*]

* Correspondence: nwqone@gmail.com

†Ling Wang and jiaxi Tang contributed equally to this manuscript.

1 Department of Phase I Clinical Trial Ward, Chongqing Key Laboratory of Translational Research for Cancer Metastasis and Individualized Treatment, Chongqing University Cancer Hospital, Chongqing 400030, China.

2 Department of Anesthesiology, Chongqing Key Laboratory of Translational Research for Cancer Metastasis and Indvidualized Treatment, Chongqing University Cancer Hospital, Chongqing 400030, China

Abstract

Introduction As an important immunotherapy, tumor vaccine has increasingly shown its important value in the treatment of malignant tumors. Neoantigens which are derived from somatic mutations of tumors and are different from normal cells have been considered as ideal vaccine targets in recent years. Individualized neoantigen vaccine developed on the

For peer review only - http://bmjopen.bmj.com/site/about/guidelines.xhtml

basis of sequencing is expected to be an important breakthrough of precision therapy for tumors.The trial aims to design and synthesize individualized tumor peptides, and evaluate the safety and efficacy of individualized tumor peptide therapy in the treatment of genomic unstable advanced solid malignant tumors.

Methods and analysis This is a non-randomized, open, non-controlled, China, single-arm, investigator initiated trial. Patients with genomic unstable advanced ovarian cancer, bladder cancer, breast cancer, cervical cancer, endometrial cancer, melanoma tumors, and digestive malignant tumors for which there is no corresponding recommended treatment in the guidelines at the time of diagnosis are eligible for study participation. A maximum of 20 patients will be included in the trial. Through the whole exome and transcriptome sequencing analysis of the fresh blood and tumor tissues of the enrolled patients, the 20 25-33aa antigen peptides with the highest mutation scores of the patients were screened out, and the corresponding new antigen peptides were synthesized and prepared. Patients will be treated with their own individualized neoantigen polypeptide combined with a polypeptide adjuvant (human granulocyte-macrophage colony stimulating factor, GM-CSF).The primary efficacy endpoint is safety. General and specific adverse events are monitored continuously. Secondary endpoints are progression-free survival （PFS）, objective response rate (ORR), objective duration of remission (DOR), 1-

For peer review only - http://bmjopen.bmj.com/site/about/guidelines.xhtml

year survival rate, and overall survival（OS）.

Ethics and dissemination This study has received approval from the Ethics Committee of Chongqing University Cancer Hospital on Nov. 21, 2019 (207/2019). Agency and is endorsed by the National Health Service. The findings of this trial will be disseminated through national and international presentations and peer-reviewed publications.

Strengths and limitations of this study

► The study will answer the question:'Whether individualized neoantigens are safe to use in human therapy?'

► The study aims to design and synthesize of individualized neoantigen tumor peptides that may lead to new treatments for cancer patients.

► The study has clearly defined the entry and exclusion criteria for inclusion in this clinical trial.

► Rigorous AE observation and follow-up provides opportunities to assess patients' safety, allowing for early trial termination if necessary.

► The trial includes low and moderate risk recipients only—the suitability for high-risk recipients will require further testing.

Trial registration ChiCTR1900025364

Introduction

Miraculous effects in the treatment of malignant tumors using neoantigens

For peer review only - http://bmjopen.bmj.com/site/about/guidelines.xhtml

Malignant tumors are a serious threat to human life and health and have become the second leading cause of human death [1]. At present, many tumors cannot be cured by conventional methods such as surgery, radiotherapy and chemotherapy. With the rapid development of biotechnology and the in-depth study of the molecular mechanisms of tumor occurrence and development, biotherapy has become the fourth mode of comprehensive tumor treatment and has received more and more attention.

Tumor biotherapy is a new therapy that applies modern biotechnology and its products for tumor prevention and treatment. It achieves anti-tumor effects by mobilizing the host's natural defense mechanism or giving natural (or genetic engineering) highly targeted substances. It mainly includes somatic cell therapy, cytokine therapy, tumor peptides therapy, molecular targeted therapy, radioimmune targeted therapy, tumor gene therapy and biological chemotherapy. Among them, tumor peptides study is one of the hotspots in domestic and foreign research in recent years. Individualized neoantigen tumor immunotherapy has brought inspiring therapeutic effects. From the Harvard University Dana-farber Cancer Center (Catherine Wu team), 6 cases of melanoma patients were treated with neoantigen peptides, and the effect was very significant. Among them, 4 cases of tumors did not progress, and 2 cases of tumors disappeared [2]. Sonntag et al. reported a case of advanced pancreatic cancer with lung

For peer review only - http://bmjopen.bmj.com/site/about/guidelines.xhtml

metastasis. The patient received neoantigen peptide therapy combined with chemotherapy for 4 years. His tumor disappeared completely and he had survived for 6 years when the article was published.[3].In addition, neoantigen therapy for bile duct epithelial cancer and colorectal cancer also showed a high rate of inhibition of tumor growth. Most tumors did not progress, and some tumors shrank or even disappeared [4].

Rationale for immunotherapy using neoantigens

Neoantigens are a series of immunogenic substance derived from tu mour-specific mutations, instead of from the normal human genome [5-8], which presented on the surface of tumor cells by major histoc ompatibility complex (MHC). The new antigen can be transferred into the patient in the form of synthetic long peptide (SLY), Ribose Nucleic Acid (RNA), activated dendritic cell (DC) and deoxyribonuc leic acid (DNA). Neoantigens are not present in normal cells, so the immune system can recognize them as “non-self” antigens [9]. The principle is to eliminate or control tumors by activating the pat ient’s own immune system [10]. In the body, neoantigenic epitopes can bind to MHC I to activate CD8+ T lymphocytes, which can di fferentiate CD8+ T lymphocytes into cytotoxic T lymphocytes (CTL) and exert cytotoxic effects. Also, neoantigenic epitopes can combine d with MHC Ⅱ to activate CD4+ T lymphocytes to differentiate int o Helper T cells, such as Th1 and Th2 cells, which interact with

For peer review only - http://bmjopen.bmj.com/site/about/guidelines.xhtml

macrophages and B lymphocytes, respectively[11, 12]. In addition, the recent popular immune checkpoint inhibitors therapy, such as progra me-d cell death protein 1 (PD-1) and programmed cell death protein lig an-d 1 (PD-L1), also activate T cells to kill tumor cells by acting on tumor neoantigens [13-17].

Methods

Study design overview

This is an open-label, non-randomised, prospective, single-arm trial, which will be conducted at Phase I Trial Ward of Tertiary Class A Cancer Hospital.

Objectives

The study aims to design and synthesize of individualized neoantigen tumor peptides. The efficacy and safety of individualized neoantigen tumor peptides therapy also will be evaluated.

Endpoints

The primary endpoint is to observe and record all adverse reactions (AE) and the incidence of serious adverse reactions (SAE) of grade 3 or above associated with the study drug within 24 weeks after the administration of the individualized neoantigen tumor polypeptide(Refer to National Cancer Institute Common Terminology Criteria for Adverse Events (NCI CTCAE)version 4.03). After that, the subjects will continue to be followed

For peer review only - http://bmjopen.bmj.com/site/about/guidelines.xhtml

at the preset follow-up points for safety. Secondary endpoints are to evaluate the PFS, ORR, DOR, 1-year survival rate, and OS (based on Enhanced computed tomography/magnetic resonance imaging (CT/MRI)). The efficiency of neoantigen peptides in inducing specific anti-tumor immunity is assessed from peripheral blood samples (based on IFN-γ, CD4+ T cells, CD8+ T cells determined by Flow Cytometry and enzyme-linked immuneospot (ELISPOT) technique). In addition, the efficacy evaluation also includes the detection of circulating tumor DNA (ctDNA) and the quality of life of cancer patients.

Additional scientific program

Genome sequencing

Whole exome and transcriptome sequencing will be tested on the patient's biopsy or surgically removed tumor tissue, and whole exome sequencing will be performed on the patient's blood sample. This gene sequencing can obtain comprehensive genomic information such as the mutations of all the patients' genes, human leukocyte antigen (HLA) genotypes, and expression levels of mutated genes, which can be used to confirm patient's entry criteria and synthesize neoantigen tumor peptides.

Design and synthesize of neoantigen tumor peptides

The main body of the neoantigen design process in this study refers to the proposal published in Nature in 2018 by Neon Therapeutics and Harvard Medical School [18]. On this basis, the unique analysis technology of

For peer review only - http://bmjopen.bmj.com/site/about/guidelines.xhtml

Beijing Xinkangyuan Biotechnology Co., Ltd. is applied to further ensure the effectiveness, specificity and safety of the neoantigen design. Neoantigens. Beijing Neoantigen Biotechnology Co., Ltd. owns a series of software copyrights such as NeoRQC®, NeoAlign®, NeoHLAType®, NeoCVC®, NeoVQC®, NeoANN®, NeoGEP®, NeoDsign®,and other software， which are under the NeoOneTM platform specially designed for neoantigens. They are used for gene mutation analysis, gene expression analysis, HLA typing, neoantigen design, and multi-directional quality control to ensure that the neoantigen design in this study meets the world's leading clinical requirements. The whole design process is shown in Fig.1.

By analyzing the whole exome and transcriptome sequencing-specific somatic mutations of fresh blood samples and tumor tissues of the enrolled patients, 20 antigen peptides sequences of 25-31aa with the highest mutation scores were screened out. According to the above-mentioned designed individualized neoantigen polypeptide sequence, the Fmoc solid-phase method was used to synthesize the polypeptide. "Ninhydrin color reaction" is used for quality control; the crude peptide is purified by High Performance Liquid Chromatography (HPLC) method; the quality of peptide is mainly checked by MS and HPLC, and the judgment is based on the correct MS, and the HPLC purity is above 95%. Individualized neoantigen tumor peptides for the treatment of genomic unstable solid tumors use 10-20 peptide mixtures. According to the Good Manufacturing

For peer review only - http://bmjopen.bmj.com/site/about/guidelines.xhtml

Practice (GMP) standard of the raw material drug, the peptides were divided into vials and freeze-dried.

Inclusion criteria

1. gender is not limited
2. patients ranged in age from 18 to 70years old
3. patients who have progressed after comprehensive treatment and diagnosed with grade 3/4 (WHO) genomic unstable(TMB>10 or MS-H) solid malignant tumors, and lack further effective treatment, or the guidelines at the time of diagnosis of the patient have no corresponding recommended treatment plan are eligible
4. patient has at least one lesion can be assessed.

Exclusion criteria

1. patients with history for hypersensitivity for the ingredients of testing drugs
2. patients with a previous history of gene therapy or any history of anti-tumor therapy in the last 4 weeks
3. female patients who are pregnant or suspected of being pregnant, or those desiring pregnancy during study period
4. lactating female patients
5. patients who were judged to be inappropriate as study subjects.
6. patients who lack of adequate tumor tissue and peripheral blood samples for genetic sequencing

For peer review only

For peer review only - http://bmjopen.bmj.com/site/about/guidelines.xhtml

Termination criteria

Patients can request withdrawal from the clinical trial at any time. For the following reasons, when it becomes difficult to continue to administer the testing drug to the subject, the responsible physician or the shared medical doctor will decide to discontinue administration of the testing drug.

1. when it is found that it does not meet the selection criteria or conflicts with exclusion criteria
2. uncontrollable T cell proliferation
3. serious adverse reactions, regardless of whether it is related to treatment
4. severe allergic reactions
5. tumor progression.

Study intervention and timeline of the study

Each patient was prepared with an individualized tumor peptide consisting of 20 antigenic peptides for subcutaneous injection. The benchmark dose is 0.3mg for each antigen peptide, and the single injection dose is 0.3mg×20=6.0mg. GM-CSF is selected as a polypeptide adjuvant, with the dosage of 0.5 mcg/kg/dose.

The medication combine individualized tumor antigen polypeptides with GM-CSF, which will be injected subcutaneously through the lower edge of the upper arm deltoid muscle. The immunotherapy injection cycle is divided into two phases: initial immunization injection and booster immunization injection, which are completed in days 1, 4, 8, 15, 22, and

For peer review only - http://bmjopen.bmj.com/site/about/guidelines.xhtml

weeks 12, 20. During the initial injection of individualized neoantigen tumor peptides, adverse reactions were recorded on the day of injection. During booster immunization, adverse reactions were recorded for 3 consecutive days after each injection. The efficacy of immunotherapy was evaluated at 4, 8, 12, 24, 36, and 48 weeks. The timeline is displayed in Fig. 2.

The individual follow-up for each participant is 12 months. Patients may enter in a succeeding phase (extended follow-up) according to the preset standards. The trial started in Q3/2019 and will last about 5 years.

Assessment of therapeutic safety and efficacy

Observe and record all AEs associated with the study drug within 24 weeks after the administration of the individualized neoantigen tumor polypeptide. Adverse reactions that require close attention during the trial include: cytokine storm-related acute inflammatory response syndrome (CRS), injection allergic reactions, neurotoxicity and systemic infections.

Evaluate the PFS, ORR, and DOR by CT/MRI according to the National Cancer Institute (NCI) criteria [19]. Quality of life using the European Organization for Research and Treatment of Cancer Quality of Life Questionnaire (EORTC QLQ-C30) [20]. In order to evaluate the efficiency of neoantigen peptides in inducing specific anti-tumor immunity, by collecting 2ml of peripheral blood samples, using flow cytometry to detect the intracellular IFN-γ, extracellular CD4+, CD8+ antigen signals of

For peer review only

For peer review only - http://bmjopen.bmj.com/site/about/guidelines.xhtml

Peripheral Blood Mononuclear Cell (PBMC), and compare and analyze the IFN- before and after the immunization injection. Changes in the number of γ, CD4+ T cells, and CD8+ T cells. IFN-γ ELISPOT technology was used to detect the changes in the number of T cells in PBMC that can be activated by new antigens and secrete IFN-γ before and after treatment. In addition, the efficacy evaluation also includes the detection of ctDNA before and after neoantigen immunotherapy.

The observed indicators include: Physical examination, vital signs, Eastern Cooperative Oncology Group (ECOG) score, weight, tumor patient quality of life scale, blood routine, urine routine, liver and kidney function, coagulogram, tumor markers. During the 8th week, 12th week, 24th week, 36th week, 48th week of the study period, enhanced CT or MRI scanning was selected to evaluate the therapeutic effect according to the target lesion site of the patients. Other observation indicators: lymphocyte subsets and cytokines secreted by cells: ctDNA; the antigen-specific lymphocyte count in patients was determined by ELISPOT technique. The intervention and evaluation schedule is shown in Table1.

For peer review only - http://bmjopen.bmj.com/site/about/guidelines.xhtml

Table 1 Intervention and assessment schedule for the INP Trial

Execute project	W-4~W0 Screening period	W1	W2	W3	W4	W5	W6	W8	W10	W12	W20	W24	W36	W48
Sequencing /Neoantigen preparation	√													
Neoantigen immunization		√ √	√	√	√					√	√			
Physical examination	√	√	√	√	√	√	√	√	√	√	√	√	√	√
Laboratory examination	√ *				√			√		√	√	√	√	√
CT/MRI	√ *							√		√		√	√	√
ctDNA detection	√ *				√			√		√		√	√	√
IFN-γ, CD4+ T cells, CD8+ T cells	√ *				√						√			√
AEs(CTCAE)	√ *	√	√	√	√	√	√	√	√	√	√	√	√	√
QLQ(EORTC QLQ-C30)	√ *				√			√		√		√		√
Survival follow-up		√	√	√	√	√	√	√	√	√	√	√	√	√

* Need to screen out qualified neoantigens before relevant testing, √√ Twice a week; *CT* computed tomography, *MRI* magnetic resonance imaging, *AE* adverse event, *CTCAE* Common Terminology Criteria for Adverse Events, *QLQ* Quality of Life Questionnaire.

For peer review only

sample size

To the best of our knowledge, there are no objective data on the efficacy of individualized neoantigens in advanced tumors.This study will not stipulate a sample size a priori. The sample size was not therefore determined by power calculations. The purpose of the trial is to evaluate the safety of individualized neoantigens, similar to the phase I clinical trials of drugs, so our sample size is determined to be 20 patients.

Statistical considerations

First, we need to list the number of selected and completed cases, confirm three data sets: Full analysis set （FAS）, per-protocol set (PPS), Safety data set (SS)， and then describe demographic data and other baseline characteristic values.

Calculate the percentage of subjects with medication compliance within the range of 80% to 120%, calculate the percentage of subjects with combined medications, and perform combined medication classification analysis. Adverse events were coded according to the ICH International Dictionary of Medical Terms (MedDRA). List the adverse events/reactions, serious adverse events/reactions, important adverse events, and adverse events/reactions that led to detachment, and calculate the incidence. Clinical ORR and 1-year survival rate were analyzed using actual data in FAS. Experimental data are assessed by using methods of descriptive data

For peer review only

For peer review only - http://bmjopen.bmj.com/site/about/guidelines.xhtml

analysis.

Patient and Public Involvement

Patients and the public will understand the design and benefits of our trial through the recruitment information and consulation of patient advisers. The patients do not participate in the design of the experiment. The results of the experiment will be kept confidential to the patients.

Ethics

The procedures set out in this trial protocol are designed to ensure that all persons involved in the trial abide by the International Conference on Harmonization (ICH) harmonized tripartite guideline on Good Clinical Practice (ICH-GCP) and the ethical principles described in the applicable version of the Declaration of Helsinki. The trial will be carried out in keeping with local legal and regulatory requirements. The regulations of the GCP regulations will be respected. Before the start of the trial, all documents were submitted to the independent ethics committee. The local ethics committee approved this study on Nov 21, 2019 (207/2019).

Data quality assurance

The histologic diagnosis must be centrally verified by one of the experienced pathologists. Tumor grading and evaluation of post-treatment curative effect should be decided by public consultation with experienced clinicians and radiologists. According to the Clinical Trial Quality Management Practice Guidelines (CPMP/ICH/135/95), we need to follow

For peer review only - http://bmjopen.bmj.com/site/about/guidelines.xhtml

the written operating standard procedures (SOP). During the clinical trial process, the supervisor should send the case report form (CRF) to the data management unit in real time, and the data administrator should perform independent double entry and double check. Inconsistent results in the entry should be checked and corrected item by item against the CRF.

Trial status

Protocol version 1.2 (27.09.2019). The trial started in September 2019 and is currently recruiting. The length of clinical phase is about 60 months. (The planned end of the study is the end of 2024.)

Discussion

Tumor neoantigens are derived from somatic cell mutations, which are present in various tumors, and there is a strong heterogeneity among them. Trials of personalized vaccines based on emergent antigens were first reported in clinical patients by Professor Carreno of the University of Washington. Their data also showed that the vaccine increased the breadth and diversity of neonatal antigen-specific T cells. Although the trial did not report the clinical therapeutic effect of the neoantigen vaccine, it proved that the neoantigen vaccine is safe and no side effects related to autoimmune diseases were found [21].

The most prominent feature of the T-cell-based immune response is its

For peer review only - http://bmjopen.bmj.com/site/about/guidelines.xhtml

memorability, which also reflects the long-term and sustained clinical effects that can be achieved in tumor immunotherapy. The 10-year follow-up data of surgically removed pancreatic cancer patients who received K-ras mutation neonatal vaccine treatment showed that their 10-year survival rate was 20% (4/20), while the 10-year survival rate of the non-vaccine treatment group was 0 (0 /87)[22]. The study by Schreiber's team at the University of Washington emphasized the key role of MHC-II neoantigen in Immune checkpoint inhibitor (ICB) immunotherapy and therapeutic tumor vaccines. The Th1 polarized immune response induced by MHC-II neoantigen plays a role in Cytotoxic T lymphocytes (CTL) activation, multiple links such as increase antitumor CTL anti-tumor ability [23].

The personalized neoantigen vaccine based on high-throughput sequencing has the advantages of precise identification and precise killing of tumor cells, which is expected to be an important breakthrough for vaccines towards clinical practice. Therefore, neoantigens can be specific targets for precision immunotherapy [24]. The identification of neoantigens and the development of neoantigens vaccine may provide more options for clinical immunotherapy. It may become one of the effective ways to treat malignant tumors in the future. The aim of this trial is to assess the safety and efficacy of neoantigens in cancer patients and explore the potential Immune effect of individual neoantigenic tumor peptides in tumor patients. Additional information can be found online at the SPIRIT 2013 checklist

For peer review only - http://bmjopen.bmj.com/site/about/guidelines.xhtml

see Additional file 1.

Ethics and dissemination

Research ethics approval

This study has received approval from the Ethics Committee of Chongqing University Cancer Hospital on Nov. 21, 2019 (207/2019). The study protocol, informed consent form and other submitted documents were reviewed and approved. The trial registration number is ChiCTR1900025364 (http://www.chictr.org.cn/index.aspx).

Confidentiality

During the clinical trial, subjects will be identified solely by means of their individual identification code. Trial data stored on a computer will be stored in accordance with local data protection law and will be handled in strictest confidence.

Dissemination policy

Regardless of the results of the research, the final data will be publicly released. After the data collection is completed (within 12 months of the end of the study), a report with the results of the research will be submitted in an appropriate open-access, peer-reviewed medical journal.

Additional file

Additional file 1: SPIRIT (Standard Protocol Items: Recommendations for Interventional Trials) 2013 Checklist: Recommended items to address in a clinical trial protocol and related documents. (DOC 236 kb)

Acknowledgments

For peer review only - http://bmjopen.bmj.com/site/about/guidelines.xhtml

This work was supported by Beijing Neoantigen Biotechnology Co., Ltd.in terms of technology. We acknowledge financial support by Special Project for Performance Incentive and Guidance of Scientific Research Institutions of Chongqing, China grant to LW, by Special Project for Performance Incentive and Guidance of Scientific Research Institutions of Chongqing, China grant to XC and by Natural Science Foundation of Chongqing, China grant to JXT. We also thank the patient advisers for their efforts throughout the study.

Contributors XC, WYT, JZ and BL developed and planned this trial, and WQN is the principal investigator of the study. LW performed basic research and wrote the manuscript. JXT is responsible for statistical planning and statistical analysis. All authors read and approved the final manuscript.

Funding
The trial is sponsored by Special Project for Performance Incentive and Guidance of Scientific Research Institutions of Chongqing, China, No. cstc2019jxjl130029, by Special Project for Performance Incentive and Guidance of Scientific Research Institutions of Chongqing, China, No. cstc2018jxjl130057 and by Natural Science Foundation of Chongqing, China, No. cstc2019jcyj-msxmX0623.

Competing interests The authors declare that they have no competing interests.

Ethics approval Ethics Committee of Chongqing University Cancer Hospital.

Provenance and peer review Not commissioned, externally peer reviewed.

Word Count 3718 words

References

[1] Heron M. Deaths: Leading Causes for 2017. Natl Vital Stat Rep. 2019. 68(6): 1-77.
[2] Ott PA, Hu Z, Keskin DB, et al. An immunogenic personal neoantigen vaccine for patients with melanoma. Nature. 2017. 547(7662): 217-221.
[3] Sonntag K, Hashimoto H, Eyrich M, et al. Immune monitoring and TCR sequencing of CD4 T cells in a long term responsive patient with metastasized pancreatic ductal carcinoma treated with individualized, neoepitope-derived multipeptide vaccines: a case report. J Transl Med. 2018. 16(1): 23.
[4] Tran E, Turcotte S, Gros A, et al. Cancer immunotherapy based on mutation-specific CD4+ T cells in a patient with epithelial cancer. Science. 2014. 344(6184): 641-5.
[5] Liu XS, Mardis ER. Applications of Immunogenomics to Cancer. Cell. 2017.

For peer review only - http://bmjopen.bmj.com/site/about/guidelines.xhtml

168(4): 600-612.
[6] Tran E, Robbins PF, Rosenberg SA. 'Final common pathway' of human cancer immunotherapy: targeting random somatic mutations. Nat Immunol. 2017. 18(3): 255-262.
[7] Vormehr M, Diken M, Boegel S, Kreiter S, Türeci Ö, Sahin U. Mutanome directed cancer immunotherapy. Curr Opin Immunol. 2016. 39: 14-22.
[8] Schumacher TN, Schreiber RD. Neoantigens in cancer immunotherapy. Science. 2015. 348(6230): 69-74.
[9] Lu YC, Robbins PF. Cancer immunotherapy targeting neoantigens. Semin Immunol. 2016. 28(1): 22-7.
[10] Quandt J, Schlude C, Bartoschek M, et al. Long-peptide vaccination with driver gene mutations in p53 and Kras induces cancer mutation-specific effector as well as regulatory T cell responses. Oncoimmunology. 2018. 7(12): e1500671.
[11] Chen X, Yang J, Wang L, Liu B. Personalized neoantigen vaccination with synthetic long peptides: recent advances and future perspectives. Theranostics. 2020. 10(13): 6011-6023.
[12] Sahin U, Türeci Ö. Personalized vaccines for cancer immunotherapy. Science. 2018. 359(6382): 1355-1360.
[13] Postow MA, Chesney J, Pavlick AC, et al. Nivolumab and ipilimumab versus ipilimumab in untreated melanoma. N Engl J Med. 2015. 372(21): 2006-17.
[14] Zou W, Wolchok JD, Chen L. PD-L1 (B7-H1) and PD-1 pathway blockade for cancer therapy: Mechanisms, response biomarkers, and combinations. Sci Transl Med. 2016. 8(328): 328rv4.
[15] Parmiani G, De Filippo A, Novellino L, Castelli C. Unique human tumor antigens: immunobiology and use in clinical trials. J Immunol. 2007. 178(4): 1975-9.
[16] Shankaran V, Ikeda H, Bruce AT, et al. IFNgamma and lymphocytes prevent primary tumour development and shape tumour immunogenicity. Nature. 2001. 410(6832): 1107-11.
[17] McGranahan N, Furness AJ, Rosenthal R, et al. Clonal neoantigens elicit T cell immunoreactivity and sensitivity to immune checkpoint blockade. Science. 2016. 351(6280): 1463-9.
[18] Keskin DB, Anandappa AJ, Sun J, et al. Neoantigen vaccine generates intratumoral T cell responses in phase Ib glioblastoma trial. Nature. 2019. 565(7738): 234-239.
[19] Cheson BD, Horning SJ, Coiffier B, et al. Report of an international workshop to standardize response criteria for non-Hodgkin's lymphomas. NCI Sponsored International Working Group. J Clin Oncol. 1999. 17(4): 1244.
[20] Aaronson NK, Ahmedzai S, Bergman B, et al. The European Organization for Research and Treatment of Cancer QLQ-C30: a quality-of-life instrument for use in international clinical trials in oncology. J Natl Cancer Inst. 1993. 85(5): 365-76.
[21] Carreno BM, Magrini V, Becker-Hapak M, et al. Cancer immunotherapy. A dendritic cell vaccine increases the breadth and diversity of melanoma neoantigen-specific T cells. Science. 2015. 348(6236): 803-8.
[22] Wedén S, Klemp M, Gladhaug IP, et al. Long-term follow-up of patients with

For peer review only - http://bmjopen.bmj.com/site/about/guidelines.xhtml

resected pancreatic cancer following vaccination against mutant K-ras. Int J Cancer. 2011. 128(5): 1120-8.

[23] Alspach E, Lussier DM, Miceli AP, et al. MHC-II neoantigens shape tumour immunity and response to immunotherapy. Nature. 2019. 574(7780): 696-701.

[24] Yarchoan M, Johnson BA 3rd, Lutz ER, Laheru DA, Jaffee EM. Targeting neoantigens to augment antitumour immunity. Nat Rev Cancer. 2017. 17(4): 209-222.

Figure Legends

Fig. 1 Design flow chart of neoantigens. *Abbreviations*: *DNA* deoxyribonucleic acid, *RNA* ri bonucleic acid, *HLA* human leucocyte antigen, *QC* quality control

Fig.2 The study flow chart

For peer review only

For peer review only - http://bmjopen.bmj.com/site/about/guidelines.xhtml

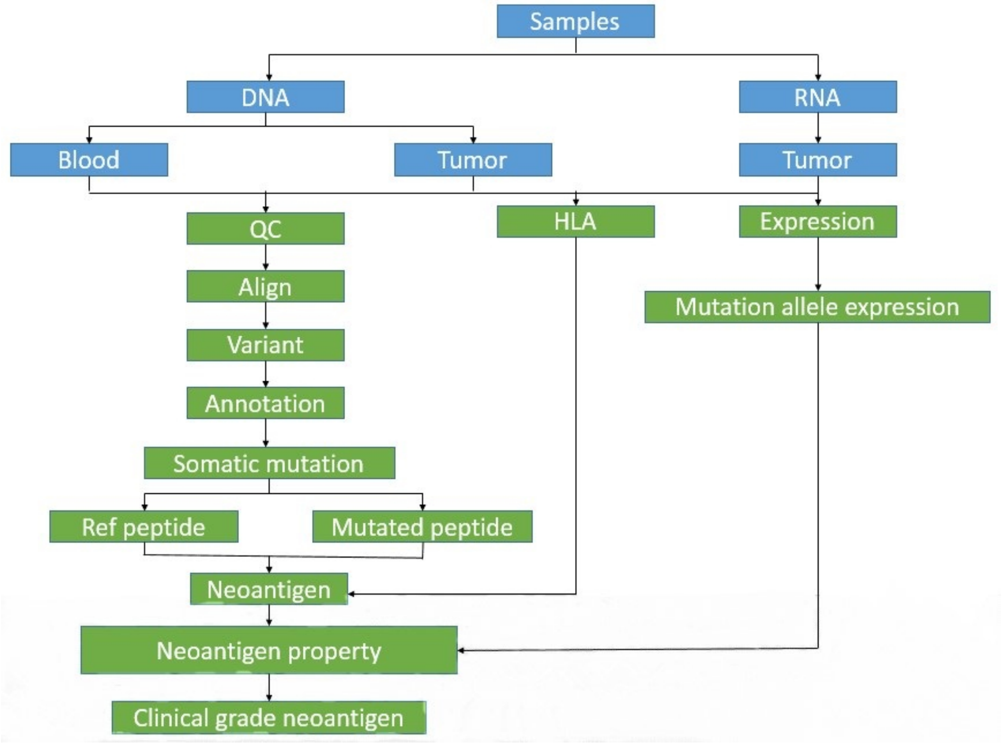

Fig. 1 Design flow chart of neoantigens. Abbreviations: DNA deoxyribonucleic acid, RNA ri bonucleic acid, HLA human leucocyte antigen, QC quality control

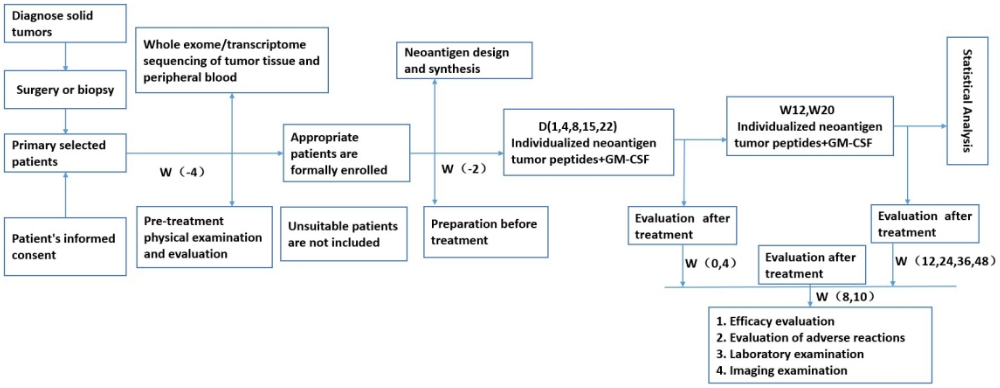

Fig.2 The study flow chart

Reporting checklist for protocol of a clinical trial.

Based on the SPIRIT guidelines.

Instructions to authors

Complete this checklist by entering the page numbers from your manuscript where readers will find each of the items listed below.

Your article may not currently address all the items on the checklist. Please modify your text to include the missing information. If you are certain that an item does not apply, please write "n/a" and provide a short explanation.

Upload your completed checklist as an extra file when you submit to a journal.

In your methods section, say that you used the SPIRITreporting guidelines, and cite them as:

Chan A-W, Tetzlaff JM, Gøtzsche PC, Altman DG, Mann H, Berlin J, Dickersin K, Hróbjartsson A, Schulz KF, Parulekar WR, Krleža-Jerić K, Laupacis A, Moher D. SPIRIT 2013 Explanation and Elaboration: Guidance for protocols of clinical trials. BMJ. 2013;346:e7586

For peer review only

		Reporting Item	Page Number
**Administrative information**			
Title	#1	Therapy of genomic unstable solid tumors（WHO grade 3/4）in clinical stage III/IV using individualized neoantigen tumor peptides - INP Trial (Individualized neoantigen tumor peptides Immunotherapy):study protocol for an open-label, non-	1

		randomised, prospective, single-arm trial	
Trial registration	#2a	ChiCTR1900025364	3
Trial registration: data set	#2b	N/A	
Protocol version	#3	1.2 (27.09.2019)	16
Funding	#4	The trial is sponsored by Special Project for Performance Incentive and Guidance of Scientific Research Institutions of Chongqing, China, No. cstc2019jxjl130029, by Special Project for Performance Incentive and Guidance of Scientific Research Institutions of Chongqing, China, No. cstc2018jxjl130057 and by Natural Science Foundation of Chongqing, China, No. cstc2019jcyj-msxmX0623.	19
Roles and responsibilities: contributorship	#5a	**Authors:**	1
		Ling Wang[1] jiaxi Tang[2] , Xia Chen[1], Juan Zhao[1], Wanyan Tang[1], Bin Liao[1], Weiqi Nian[1]	
		**Affiliations:**	1
		1 Department of Phase I Clinical Trial Ward, Chongqing Key Laboratory of Translational Research for Cancer Metastasis and Individualized Treatment, Chongqing University Cancer Hospital, Chongqing 400030, China	

For peer review only

		2 Department of Anesthesiology, Chongqing Key Laboratory of Translational Research for Cancer Metastasis and Indvidualized Treatment, Chongqing University Cancer Hospital, Chongqing 400030, China	
		**roles of protocol contributors：**	19
		XC, WYT, JZ and BL developed and planned this trial, and WQN is the principal investigator of the study. LW performed basic research and wrote the manuscript. JXT is responsible for statistical planning and statistical analysis. All authors read and approved the final manuscript.	
Roles and responsibilities: sponsor contact information	#5b	Weiqi Nian，Department of Phase I Clinical Trial Ward, Chongqing Key Laboratory of Translational Research for Cancer Metastasis and Individualized Treatment, Chongqing University Cancer Hospital, No. 181 Hanyu Road, Shapingba District, Chongqing 400030, China. nwqone@gmail.com	1
Roles and responsibilities: sponsor and funder	#5c	Research sponsors and funders participate in the role of research design, LW performed basic research and wrote the manuscript. JXT is responsible for statistical planning and statistical analysis. CX is responsible for planning and developing the trial, and they jointly determine the decision to submit the report for publication, and they have the final decision on these activities,	19
Roles and responsibilities: committees	#5d	XC, WYT, YG and CC developed and planned this trial, and WQN is the principal investigator of the study. LW performed basic research and wrote the manuscript. JXT is responsible for statistical planning and statistical analysis. All authors read and approved the final manuscript.	19

For peer review only

**Introduction**			
Background and rationale	#6a	manuscript pages 3-6	3-6
Background and rationale: choice of comparators	#6b	N/A	
Objectives	#7	The study aims to design and synthesize of individualized neoantigen tumor peptides. The efficacy and safety of individualized neoantigen tumor peptides therapy also will be evaluated.	6
Trial design	#8	This is an open-label, non- randomised, prospective, single-arm trial	6
**Methods: Participants, interventions, and outcomes**			
Study setting	#9	Phase I Trial Ward of Tertiary Class A Cancer Hospital	6
Eligibility criteria	#10	**Inclusion criteria**<br>1. gender is not limited<br>2. patients ranged in age from 18 to 70years old<br>3. patients who have progressed after comprehensive treatment and diagnosed with grade 3/4 (WHO) genomic unstable(TMB>10	9-10

For peer review only

		or MS-H) solid malignant tumors, and lack further effective treatment, or the guidelines at the time of diagnosis of the patient have no corresponding recommended treatment plan are eligible<br><br>4. patient has at least one lesion can be assessed.<br><br>**Exclusion criteria**<br><br>1. patients with history for hypersensitivity for the ingredients of testing drugs<br><br>2. patients with a previous history of gene therapy or any history of anti-tumor therapy in the last 4 weeks<br><br>3. female patients who are pregnant or suspected of being pregnant, or those desiring pregnancy during study period<br><br>4. lactating female patients<br><br>5. patients who were judged to be inappropriate as study subjects.<br><br>6. patients who lack of adequate tumor tissue and peripheral blood samples for genetic sequencing	
Interventions: description	#11a	Each patient was prepared with an individualized tumor peptide consisting of 20 antigenic peptides for subcutaneous injection. The benchmark dose is 0.3mg for each antigen peptide, and the single injection dose is 0.3mg×20=6.0mg. GM-CSF is selected as a polypeptide adjuvant, with the dosage of 0.5 mcg/kg/dose.<br><br>The medication combine individualized tumor antigen polypeptides with GM-CSF, which will be injected subcutaneously through the	10-11

For peer review only

		lower edge of the upper arm deltoid muscle. The immunotherapy injection cycle is divided into two phases: initial immunization injection and booster immunization injection, which are completed in days 1, 4, 8, 15, 22, and weeks 12, 20. During the initial injection of individualized neoantigen tumor peptides, adverse reactions were recorded on the day of injection. During booster immunization, adverse reactions were recorded for 3 consecutive days after each injection. The efficacy of immunotherapy was evaluated at 4, 8, 12, 24, 36, and 48 weeks. The individual follow-up for each participant is 12 months. Patients may enter in a succeeding phase (extended follow-up) according to the preset standards. The trial started in Q3/2019 and will last about 5 years.<br><br>The timeline is displayed in Fig. 2.	
Interventions: modifications	#11b	Patients can request withdrawal from the clinical trial at any time. For the following reasons, when it becomes difficult to continue to administer the testing drug to the subject, the responsible physician or the shared medical doctor will decide to discontinue administration of the testing drug.<br><br>1. when it is found that it does not meet the selection criteria or conflicts with exclusion criteria<br><br>2. uncontrollable T cell proliferation<br><br>3. serious adverse reactions, regardless of whether it is related to treatment<br><br>4. severe allergic reactions	10-11

For peer review only

		5. tumor progression.	
Interventions: adherance	#11c	According to the Clinical Trial Quality Management Practice Guidelines (CPMP/ICH/135/95), we need to follow the written operating standard procedures (SOP). Quality control applies to every stage of data processing. Take the following steps to ensure the accuracy, consistency, completeness and reliability of the data ：Researcher meeting, Research center review of main efficacy variables, Routine research center monitoring, Continuous research center communication and training, Data management quality control check Continuous data acquisition and cleanup, Internal review of data, Quality control inspection of final clinical study report, Partner performs all monitoring duties. The inspectors from Beijing New Kangyuan Biotechnology Co. Ltd. will carry out their work in accordance with the SOP of Beijing New Kangyuan Biotechnology Co.Ltd. The inspector will establish and maintain regular contacts between the investigator, partner and sponsor. They will arrange the supply of test drugs to ensure that the storage conditions of the test drugs meet the requirements. The investigator should verify laboratory test results that are outside the clinically acceptable range, both before and after treatment. If they are caused by diseases other than experimental diseases or diseases not permitted to be combined, they should be reexamined in time. If they are truly abnormal, they should not be included before treatment and should be followed up to normal after treatment.	N/A

For peer review only

Interventions: concomitant care	#11d	Related additional diseases that occurred at the time of informed consent are regarded as concomitant diseases and will be recorded on the corresponding page of the case report form (CRF). The first abnormality or deterioration (intensity, frequency) in the trial is an adverse event (AEs) and must be recorded on the corresponding page of the CRF. If there is no effect on the outcome measurement used in this study and does not interfere with the trial drug, treatment of concomitant diseases that are not restricted by the exclusion criteria is allowed. If drugs are used at the same time, these must be recorded in the patient file and CRF. Adverse reactions/effects after injection of antigen peptides and adjuvants, such as fever, rash, laryngospasm, dyspnea, shock, can be treated with corresponding drugs (bupleurum, fenbid, ibuprofen, aspirin, antihistamines, Anti-allergic and anti-shock drugs). After the injection of antigen peptides and adjuvants, patients with corresponding clinical symptoms (such as systemic infection) due to hypogammaglobulinemia can be treated with gamma globulin. After the injection of antigen peptides and adjuvants, if acute inflammatory response syndrome related to cytokine storm occurs, give a dose of tocilizumab, which can be repeated if necessary. If life-threatening acute inflammatory response syndrome occurs, large Dosage hormones. Subjects can receive fully supportive treatment according to the treatment standards as needed.<br><br>If no serious side effects occur, adrenal glucocorticoids are not	N/A

For peer review only

		allowed. During the research and treatment process, the following treatments are not allowed: systemic anti-cancer treatment (chemotherapy, immunotherapy, stem cell transplantation treatment), no other anti-tumor drug treatment within 24 weeks after injection of antigen peptides and adjuvants, and no stem cell transplantation treatment. During the study period, subjects and sexual partners were forced to adopt appropriate contraceptive measures (contraceptives, condoms, intrauterine contraceptive rings, surgical methods or abstinence). Breastfeeding is prohibited during the treatment period and 6 months after treatment.	
Outcomes	#12	The primary endpoint is to observe and record all adverse reactions (AE) and the incidence of serious adverse reactions (SAE) of grade 3 or above associated with the study drug within 24 weeks after the administration of the individualized neoantigen tumor polypeptide(Refer to National Cancer Institute Common Terminology Criteria for Adverse Events (NCI CTCAE)version 4.03). After that, the subjects will continue to be followed at the preset follow-up points for safety. Secondary endpoints are to evaluate the PFS, ORR, DOR, 1-year survival rate, and OS (based on Enhanced computed tomography/magnetic resonance	6-7

For peer review only

		imaging (CT/MRI)). The efficiency of neoantigen peptides in inducing specific anti-tumor immunity is assessed from peripheral blood samples (based on IFN-γ, CD4+ T cells, CD8+ T cells determined by Flow Cytometry and enzyme-linked immuneospot (ELISPOT) technique). In addition, the efficacy evaluation also includes the detection of circulating tumor DNA (ctDNA) and the quality of life of cancer patients.	
Participant timeline	#13	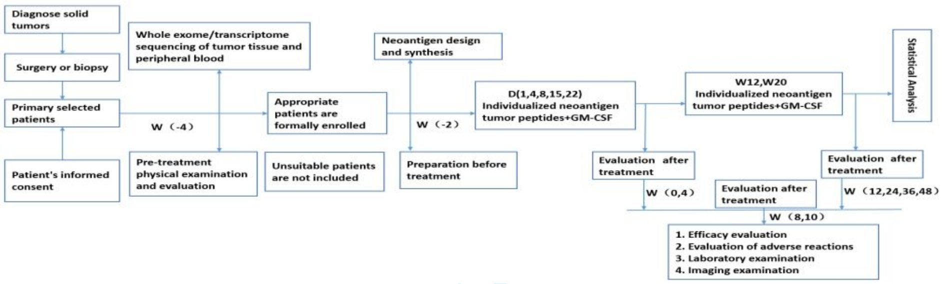	12
Sample size	#14	To the best of our knowledge, there are no objective data on the efficacy of individualized neoantigens in advanced tumors.This study will not stipulate a sample size a priori. The sample size was not therefore determined by power calculations. The purpose of the trial is to evaluate the safety of individualized neoantigens, similar to the phase I clinical trials of drugs, so our sample size is determined to be 20 patients.	14
Recruitment	#15	The hospital where the research site is located is a tertiary-A tumor specialist hospital with sufficient disease sources. In	N/A

For peer review only

		addition, the research hospital has joined a tumor alliance of 20 hospitals, which can also provide adequate and suitable disease sources.	
**Methods: Assignment of interventions (for controlled trials)**		N/A	N/A
Allocation: sequence generation	#16a	n.a., no randomization	N/A
Allocation concealment mechanism	#16b	n.a., no randomization	N/A
Allocation: implementation	#16c	n.a., no randomization	N/A
Blinding (masking)	#17a	n.a., no blinded treatment	N/A
Blinding (masking): emergency unblinding	#17b	n.a., no blinded treatment	N/A
**Methods: Data**			

For peer review only

**collection, management, and analysis**			
Data collection plan	#18a	All entries in the CRF will be verifiable by source documents. The histologic diagnosis must be centrally verified by one of the experienced pathologists. Tumor grading and evaluation of post-treatment curative effect should be decided by public consultation with experienced clinicians and radiologists. According to the Clinical Trial Quality Management Practice Guidelines (CPMP/ICH/135/95), we need to follow the written operating standard procedures (SOP).	15
Data collection plan: retention	#18b	Researchers will do their best to ensure that most subjects receive at least one tumor assessment during the treatment cycle. For the main analysis, subjects in the PPS population who withdrew from treatment due to toxicity, early disease progression, or death, in this case, the subjects will be classified as non-responders.	N/A
Data management	#19	A detailed list will be provided in the Investigator Site File. Regardless, there must be a minimum documentation, which provides information on study participation and includes all medical information necessary for appropriate medical care outside of the clinical trial in the patient record. The investigator is responsible for ensuring that all sections of the CRF are completed correctly and that entries can be verified against source data. Any errors should have a single line drawn through them so that the original entry remains legible and the correct data should	N/A

For peer review only

		be entered at the side with the investigator's signature, date and reason for change. Self-explanatory corrections need not to be justified. The correctness of entries in CRF will be confirmed by dated signature of the responsible investigator. During the clinical trial process, the supervisor should send the case report form (CRF) to the data management unit in real time, and the data administrator should perform independent double entry and double check. The original CRF will be transferred to the data management Institute of Medical Biometry and Informatics (IMBI) Chongqing; one copy will be remained by the investigator.	
Statistics: outcomes	#20a	First, we need to list the number of selected and completed cases, confirm three data sets: Full analysis set （FAS）, per-protocol set (PPS), Safety data set (SS), and then describe demographic data and other baseline characteristic values. Calculate the percentage of subjects with medication compliance within the range of 80% to 120%, calculate the percentage of subjects with combined medications, and perform combined medication classification analysis. Adverse events were coded according to the ICH International Dictionary of Medical Terms (MedDRA). List the adverse events/reactions, serious adverse events/reactions, important adverse events, and adverse events/reactions that led to detachment, and calculate the incidence. Clinical ORR and 1-year survival rate were analyzed using actual data in FAS. Experimental data are assessed by using methods of descriptive data analysis.	14

For peer review only

Statistics: additional analyses	#20b	N/A	N/A
Statistics: analysis population and missing data	#20c	N/A	N/A
**Methods: Monitoring**			
Data monitoring: formal committee	#21a	The data monitoring committee (DMC) is a group of experts with various professional knowledge and experience, independent of the sponsor and the organizations that have interests with the sponsor. The DMC is established to independently review and evaluate accumulating safety data from an ongoing clinical trial in order to protect the safety of the subjects and to evaluate the evolving risk-benefit if required.	N/A
Data monitoring: interim analysis	#21b	The trial can be stopped by the LKP if the DMC detects an unexpected accumulation of side effects (each grade 5, 2 succeeding grade 4 or 3 succeeding grade 3 CTC side effects), has new information about the effect-risk ratio of the investigated therapy or the used methods of examination. All investigators have to be informed immediately about a stopping or a permanent ending of the trial. The participating sites must accept the decision. A participating site can also be closed early by the LKP if the site	N/A

For peer review only

		does not act according to ICH-GCP or according to the trial protocol or the recruitment or the quality of the data is below the expectations. If the DMC suggests an interruption or an ending of the trial due to SAE evaluations or other reasons, the ethical board and the authorities (BOB and sate authority) have to be informed. The Ethical Board and the authorities can also decide about an interruption or an ending of the trial.	
Harms	#22	By frequent communications (letters, telephone, fax, e-mail), the site monitor will ensure that the trial is conducted according to the protocol and regulatory requirements. Frequency and details of monitoring will be defined in the monitoring manual. If there are major findings during monitoring or an audit, the investigational site might be closed by the LKP.	N/A
Auditing	#23	The sponsor appoints knowledgeable persons as the clinical trial monitors to monitor and report on the progress of the trial and to verify relevant data. The monitors will conduct an initiation visit before initiation, a follow-up visit after the first patient is enrolled, and subsequent routine monitoring every 8-12 weeks.	N/A
**Ethics and dissemination**			
Research ethics approval	#24	This study has received approval from the Ethics Committee of Chongqing University Cancer Hospital on Nov. 21, 2019 (207/2019). The study protocol, informed consent form and other submitted documents were reviewed and approved. The trial registration number is ChiCTR1900025364 (http://www.chictr.org.cn/index.aspx).	18

For peer review only

Protocol amendments	#25	The Coordinating Investigator and all investigators will be given an up-to-date investigator's brochure containing full details of the status of the pre-clinical and clinical knowledge of the study medication. As soon as new information is obtained, an updated version will be supplied or an amendment added to the existing investigator's brochure. The investigator ensures that all team members are informed adequately about the protocol, all amendments to the protocol, the study procedures und study specific duties and tasks. The investigator will maintain a list to delegate tasks to the team members.	N/A
Consent or assent	#26a	Phase I ward trial physician	N/A
Consent or assent: ancillary studies	#26b	N/A	N/A
Confidentiality	#27	During the clinical trial, subjects will be identified solely by means of their individual identification code. Trial data stored on a computer will be stored in accordance with local data protection law and will be handled in strictest confidence.	18
Declaration of	#28	The authors declare that they have no competing interests.	19

For peer review only

interests			
Data access	#29	According to ICH-GCP the investigator(s)/institution(s) must provide direct access to source data/documents for trial related monitoring, audits and regulatory inspection. Each subject has consented - via written informed consent - to direct access to his/her original medical records for trialrelated monitoring, audit and regulatory inspection. Any data to be recorded directly on the CRFs (i.e., no prior written or electronic record of data), and to be considered to be source data must be clearly identified. In the absence of either an audit-trail or limited access for the monitor the electronic record of data must be printed out (certified copy). In case of changes a new print-out has to be made. Regulatory authorities and/ or auditors authorised by the sponsor may request access to all source documents, CRFs, and other trial documentation. The investigator who must provide support at all times for these activities must guarantee direct access to these documents. The investigator will inform the sponsor immediately about a planned inspection	N/A
Ancillary and post trial care	#30	N/A	N/A
Dissemination policy: trial results	#31a	N/A	N/A
Dissemination policy:	#31b	Publication will be prepared under the lead of the principal investigator of the study. The first and last authorship are reserved	N/A

For peer review only

authorship		for the principal investigator and the study coordinator of the study if both do not wish to transfer their authorship to a third person. All data will be published independently of the results of the trial.	
Dissemination policy: reproducible research	#31c	N/A	N/A

Appendices

Informed consent materials	#32	Before being admitted to the clinical trial, the patient must consent to participate after being fully informed by the investigator or a designated member of the investigating team about the nature, importance, risks and individual consequences of the clinical trial and their right, to terminate the participation at any time. The patient should also have the opportunity to consult the investigator, or a physician member of the investigating team about the details of the clinical trial. The informed consent to participate in the clinical trial may be withdrawn by the patient verbally in the presence of, or in written form directed to, the investigator or a physician member of the investigating team at any time during the trial. The patient must not entail any disadvantage therefore or be coerced or unduly influenced to continue to participate. Furthermore, the patient is not obligated to disclose reasons for the withdrawal of the consent. If the patient has a primary physician, the investigator should inform him or her about the patient's participation in the trial, provided the patient	N/A

For peer review only

		agrees hereto. After reading the informed consent document, the patient must give consent in writing. The patient's consent must be confirmed by the personally dated signature of the patient and by the personally dated signature of the physician conducting the informed consent discussion. If the patient is unable to write, oral presentation and explanation of the content of the informed consent form and of the data protection information must take place in the presence of an impartial witness. The witness and the physician conducting the informed consent discussions must also sign and personally date the consent document. The witness must not be in any way dependent on the sponsor of the trial, the trial site or any member of the investigating team (e. g. an employee at the trial site.). A copy of the signed informed consent document must be given to the subject; the original will be filed by the investigator. The documents must be in a language understandable to the subject and must specify who informed the subject. The subjects will be informed as soon as possible if new information may influence his/her decision to participate in the trial. The communication of this information should be documented. The informed consent for genetic profiling and the extended follow-up according to a patient register will be independent of the consent of participation in the trial.	
Biological specimens	#33	Whole exome and transcriptome sequencing will be tested on the patient's biopsy or surgically removed tumor tissue, and whole exome sequencing will be performed on the patient's blood sample. This gene sequencing can obtain comprehensive	7,11,12

For peer review only

genomic information such as the mutations of all the patients' genes, human leukocyte antigen (HLA) genotypes, and expression levels of mutated genes, which can be used to confirm patient's entry criteria and synthesize neoantigen tumor peptides. In addition, the patient's peripheral blood samples need to be used to check blood routine, liver and kidney function, and coagulation function to determine whether the patient is enrolled. It is also used to judge the immune response after treatment and to check circulating tumor DNA

None The SPIRIT Explanation and Elaboration paper is distributed under the terms of the Creative Commons Attribution License CC-BY-NC. This checklist can be completed online using https://www.goodreports.org/, a tool made by the EQUATOR Network in collaboration with Penelope.ai

For peer review only

BMJ Open

Therapy of genomic unstable solid tumors（WHO grade 3/4）in clinical stage III/IV using individualized neoantigen tumor peptides-INP Trial (Individualized neoantigen tumor peptides Immunotherapy):study protocol for an open-label, non- randomized, prospective, single-arm trial

Journal:	*BMJ Open*
Manuscript ID	bmjopen-2021-055742.R1
Article Type:	Protocol
Date Submitted by the Author:	02-Apr-2022

Complete List of Authors:	Wang, Ling; Department of Phase I Clinical Trial Ward, Chongqing University Cancer Hospital.<br>Tang, jiaxi; Department of Anesthesiology, Chongqing University Cancer Hospital.<br>Chen, Xia; Department of Clinical Trial Center, Chongqing University Cancer Hospital.<br>Zhao, Juan; Department of Phase I Clinical Trial Ward, Chongqing University Cancer Hospital.<br>Tang, Wanyan; Department of Phase I Clinical Trial Ward, Chongqing University Cancer Hospital.<br>Liao, Bin; Department of Phase I Clinical Trial Ward, Chongqing University Cancer Hospital.<br>Nian, Weiqi; Department of Oncology, Chongqing Hospital of Traditional Chinese Medicine.; Department of Phase I Clinical Trial Ward, Chongqing University Cancer Hospital.

**Primary Subject Heading**:	Oncology
Secondary Subject Heading:	Immunology (including allergy), Pharmacology and therapeutics, Genetics and genomics
Keywords:	ONCOLOGY, IMMUNOLOGY, Cancer genetics < GENETICS, CLINICAL PHARMACOLOGY

SCHOLARONE™
Manuscripts

Therapy of genomic unstable solid tumors（WHO grade 3/4）in clinical stage III/IV using individualized neoantigen tumor peptides-INP Trial (Individualized neoantigen tumor peptides Immunotherapy):study protocol for an open-label, non-randomized, prospective, single-arm trial

Ling Wang[1] †, jiaxi Tang[2] †, Xia Chen[3], Juan Zhao[1], Wanyan Tang[1], Bin Liao[1], Weiqi Nian[4,1*]

* Correspondence: nwqone@gmail.com.

†Ling Wang and jiaxi Tang contributed equally to this work, equal first author.

1 Department of Phase I Clinical Trial Ward, Chongqing University Cancer Hospital.

2 Department of Anesthesiology, Chongqing University Cancer Hospital.

3 Department of Clinical Trial Center, Chongqing University Cancer Hospital.

4 Department of Oncology, Chongqing Hospital of Traditional Chinese Medicine.

Abstract

Introduction Neoantigens derived from tumor somatic mutations are recognized as ideal vaccine targets. Tumor neoantigens have been studied in a wide range of tumors. Most of research on neoantigens has focused just on a unique tumor and a single mutated gene. Currently, few studies have reported using a mixture of neoantigen peptides derived from multiple genetic mutation sites in the treatment of genomic unstable advanced solid malignancies. The trial aims to

For peer review only - http://bmjopen.bmj.com/site/about/guidelines.xhtml

evaluate the safety and efficacy of individualized tumor neoantigen peptide mixtures in the treatment of genomic unstable advanced solid malignant tumors.

Methods and analysis This is a prospective, non-randomized, open, single-center, single-arm, phase I trial. Patients with genomic unstable advanced solid malignancies are eligible for study participations. 20 patients will be included in the trial. Through the whole exome and transcriptome sequencing analysis of the fresh blood and tumor tissues of the enrolled patients, the 20 25-33aa antigen peptides with the highest mutation scores of the patients will be screened out, and the corresponding new antigen peptides will be synthesized and prepared. Patients will be treated with their own individualized neoantigen polypeptide combined with a polypeptide adjuvant (human granulocyte-macrophage colony stimulating factor, GM-CSF). The primary endpoint is Safety indicators, including general and specific adverse events (AE) which will be monitored continuously. Secondary endpoints are progression-free survival (PFS), objective response rate (ORR), objective duration of remission (DOR), 1-year survival rate, and overall survival (OS).

Ethics and dissemination This study has received approval from the Ethics Committee of Chongqing University Cancer Hospital on Nov. 21, 2019 (207/2019). The findings of this trial will be disseminated through national and international presentations and peer-reviewed publications.

For peer review only - http://bmjopen.bmj.com/site/about/guidelines.xhtml

Trial registration number ChiCTR1900025364

Strengths and limitations of this study

►In this study, the design and synthesize of individualized neoantigen tumor peptides will be completed by a professional team to ensure the quality of tumor neoantigen.

►The study has clearly defined the entry and exclusion criteria for inclusion in this clinical trial.

►Rigorous Adverse events observation and follow-up give opportunities to evaluate safety of patients, allowing for early trial intervention if necessary.

► This trial only includes subjects with a high mutation burden, and its suitability for subjects with a low mutation burden needs further research.

►Since this study is a phase I trial, the sample size is limited, so future clinical studies with a larger sample size are necessary.

Introduction

Malignant tumors are a serious threat to human life and health and have become the second leading cause of human death [1]. At present, many tumors cannot be cured by conventional methods such as surgery, radiotherapy and chemotherapy. With the rapid development of biotechnology and the in-depth study of the molecular mechanisms of

For peer review only - http://bmjopen.bmj.com/site/about/guidelines.xhtml

tumor occurrence and development, biotherapy has become the fourth mode of comprehensive tumor treatment and has received more and more attention[2-4].

Tumor biotherapy is a new therapy that applies modern biotechnology and its products for tumor prevention and treatment. It achieves anti-tumor effects by mobilizing the host's natural defense mechanism or giving natural (or genetic engineering) highly targeted substances. It mainly includes somatic cell therapy, cytokine therapy, tumor peptides therapy, molecular targeted therapy, radioimmune targeted therapy, tumor gene therapy and biological chemotherapy[3-9]. Among them, tumor neoantigen study is one of the hotspots in domestic and foreign research in recent years[10]. Individualized neoantigen tumor immunotherapy has brought inspiring therapeutic effects. Several studies have shown that tumor neoantigen therapy can produce miraculous effects, inhibiting tumor progression, and even achieving complete tumor regression[11-13].

Tumor neoantigens are a series of immunogenic substance derived from tumor-specific mutations, instead of from the normal human genome[14-17], which presented on the surface of tumor cells by major histocompatibility complex (MHC). The new antigen can be transferred into the patient in the form of synthetic long peptide, Ribose Nucleic Acid (RNA), activated dendritic cell and deoxyribonucleic acid (DNA)[18-21]. Neoantigens are not present in normal cells, so the immune system can

For peer review only - http://bmjopen.bmj.com/site/about/guidelines.xhtml

recognize them as "non-self" antigens [22]. The principle is to eliminate or control tumors by activating the patient's own immune system [20]. In the body, neoantigen epitopes can bind to MHC-Ⅰ to activate CD8+T lymphocytes, which can differentiate CD8+T lymphocytes into cytotoxic T lymphocytes (CTL) and exert cytotoxic effects. Also, neoantigen epitopes can combine with MHC-Ⅱ to activate CD4+T lymphocytes to differentiate into helper T cells, such as Th1 and Th2 cells, which interact with macrophages and B lymphocytes, respectively[23 24].

Tumor neoantigens have been studied in a wide range of tumors, including lung, breast, glioma, liver, kidney, ovarian, Pseudomyxoma peritonei and pancreatic cancer, etc[5 12 18 25-28]. Most of research on neoantigens has focused just on a unique tumor and a single mutated gene[5 11 29]. The safety and efficacy of neoantigen peptide mixtures derived from multiple gene mutation sites in the treatment of advanced solid malignancies with genomic instability are still unclear.

The trial aims to evaluate the safety and efficacy of individualized tumor neoantigen peptide mixtures in the treatment of genomic unstable advanced solid malignant tumors.

Methods

Study design overview

This is an open-label, non-randomized, prospective, single-arm trial, which

will be conducted at Phase I Trial Ward of Tertiary Class A Cancer Hospital.

Objectives

The trial aims to evaluate the safety and efficacy of individualized tumor neoantigen peptides mixtures in the treatment of genomic unstable advanced solid malignant tumors.

Endpoints

The primary endpoint is to observe and record all Adverse Events (AE) and the incidence of serious adverse events (SAE) (grade 3 or above AE) associated with the study drug within 24 weeks after the administration of the individualized neoantigen tumor polypeptide (Refer to National Cancer Institute Common Terminology Criteria for Adverse Events (NCI CTCAE) version 4.03). After that, the subjects will continue to be followed at the preset follow-up points for safety. Secondary endpoints are to evaluate the progression-free survival (PFS), objective response rate (ORR), objective duration of remission (DOR), 1-year survival rate, and overall survival (OS) (based on Enhanced computed tomography/magnetic resonance imaging (CT/MRI)). The efficiency of neoantigen peptides in inducing specific anti-tumor immunity is assessed from peripheral blood samples (based on IFN-γ, CD4+ T cells, CD8+ T cells determined by flow cytometry and enzyme-

For peer review only - http://bmjopen.bmj.com/site/about/guidelines.xhtml

linked immuneospot (ELISPOT) technique. In addition, the efficacy evaluation also includes the detection of circulating tumor DNA (ctDNA) and the quality of life of cancer patients.

Additional scientific program

Genome sequencing

Whole exome and transcriptome sequencing will be tested on the patient's biopsy or surgically removed tumor tissue, and whole exome sequencing will also be performed on the patient's blood sample. This gene sequencing can obtain comprehensive genomic information such as the mutation status of all genes, human leukocyte antigen (HLA) genotypes, and expression levels of mutated genes in the patients, which can be used to confirm patient's entry criteria and synthesize neoantigen tumor peptides.

Design and synthesize of neoantigen tumor peptides

The main body of the neoantigen design process in this study refers to the proposal published in Nature in 2018 by Neon Therapeutics and Harvard Medical School [28]. On this basis, the unique analysis technology of Beijing Xinkangyuan Biotechnology Co., Ltd. will be applied to further ensure the quality of tumor neoantigen peptides. Beijing Neoantigen Biotechnology Co., Ltd. owns a series of software copyrights such as NeoRQC®, NeoAlign®, NeoHLAType®, NeoCVC®, NeoVQC®, NeoANN®,

For peer review only - http://bmjopen.bmj.com/site/about/guidelines.xhtml

NeoGEP®, NeoDsign®, and other softwares which are under the NeoOne™ platform specially designed for neoantigen design and synthesis. They will be used for gene mutation analysis, gene expression analysis, HLA typing, neoantigen design, and multi-directional quality control to ensure that the neoantigen design in this study meets the world's leading clinical requirements. The whole design process is shown in Fig.1.

By analyzing the whole exome and transcriptome sequencing-specific somatic mutations of fresh blood samples and tumor tissues of the enrolled patients, 20 antigen peptides sequences of 25-31aa with the highest mutation scores will be screened out. According to the above-mentioned designed individualized neoantigen polypeptide sequence, the Fmoc solid-phase method will be used to synthesize the polypeptide. Ninhydrin color reaction will be used for quality control. The crude peptide will be purified by High Performance Liquid Chromatography (HPLC) method, then the quality of peptide will be checked by Mass Spectrometer (MS) and HPLC. Qualified peptides should meet two criteria: correct MS and HPLC purity above 95%. Individualized neoantigen tumor peptides for the treatment of genomic unstable solid tumors use 20 peptide mixtures. According to the Good Manufacturing Practice (GMP) standard of the raw material drug, the peptides will be separated and frozen for subsequent trials.

Inclusion criteria

For peer review only - http://bmjopen.bmj.com/site/about/guidelines.xhtml

1. Gender is not limited
2. Patients range in age from 18 to 70 years old
3. Patients who have progressed after comprehensive treatment and diagnosed with grade 3/4 (World Health Organization (WHO)) genomic unstable (tumor mutational burden (TMB) >10 or microsatellite instability-high (MSI-H)) solid malignant tumors, and lack further effective treatment, or the guidelines at the time of diagnosis of the patient have no corresponding recommended treatment plan are eligible
4. There is at least one lesion that can be evaluated.

Exclusion criteria

1. Patients who have a history of hypersensitivity to the testing drug ingredients
2. Patients with a previous history of gene therapy or any history of anti-tumor therapy in the last 4 weeks
3. Female patients who are currently pregnant, suspected of being pregnant, or expect to become pregnant during the study period
4. Patients who are judged to be inappropriate as study subjects.
5. Patients who lack sufficient tumor tissues and peripheral blood samples for gene sequencing

Termination criteria

For peer review only - http://bmjopen.bmj.com/site/about/guidelines.xhtml

Patients can request withdrawal from the clinical trial at any time. If it becomes difficult to administrate the testing drug to the subject due to the following reasons, the responsible physician or the sharing physician will decide to discontinue the drug administration.

1. Any time it is found that the patient does not meet the inclusion criteria or meets the exclusion criteria
2. Uncontrollable T cell proliferation
3. SAEs, regardless of whether they are related to treatment
4. Tumor progression.

Study intervention and timeline of the study

Individualized tumor peptide mixtures of 20 antigenic peptides will be injected subcutaneously for therapeutic intervention in patients. The benchmark dose is 0.3mg for each antigen peptide, and the single injection dose is 0.3mg×20=6.0mg. GM-CSF will be selected as a polypeptide adjuvant, with the dosage of 0.5 mcg/kg/dose.

The medicine combine individualized tumor antigen polypeptides and GM-CSF will be injected subcutaneously at the lower edge of the upper arm deltoid muscle. The immunotherapy injection cycle is divided into two phases: initial immunization injection and booster immunization injection, which are completed in days 1, 4, 8, 15, 22, and weeks 12, 20. During the initial injection of individualized neoantigen tumor peptides, AEs will be

For peer review only - http://bmjopen.bmj.com/site/about/guidelines.xhtml

recorded on the day of injection. During booster immunization, AEs will be recorded for 3 consecutive days after each injection. The efficacy of immunotherapy will be evaluated at 4, 8, 12, 24, 36, and 48 weeks. The timeline is displayed in Fig. 2.

The individual follow-up for each participant is 12 months. Patients may enter in a succeeding phase (extended follow-up) according to the preset standards. The trial started in Q3/2019 and will last about 5 years.

Assessment of therapeutic safety and efficacy

After treatment with individual neoantigen tumor polypeptides, all drug-related AEs occurring within 24 weeks will be observed and recorded. Adverse reactions that require close attention during the trial include cytokine storm-related acute inflammatory response syndrome (CRS), allergic reactions, neurotoxicity and systemic infections.

PFS, ORR, and DOR will be evaluated by CT/MRI according to the National Cancer Institute (NCI) criteria[30]. Quality of life will be assessed using the Quality-of-Life questionnaire of the European Organization for Research and Treatment (EORTC QLQ-C30)[31]. In addition, 2ml peripheral blood samples will be collected and IFN-γ, CD4+, CD8+ antigen signals in peripheral blood will be detected by flow cytometry to evaluate the effectiveness of tumor neoantigen polypeptide in inducing specific anti-tumor immunity. The changes of IFN-γ cells, CD4+T cells, and CD8+T

For peer review only - http://bmjopen.bmj.com/site/about/guidelines.xhtml

cells in the peripheral blood mononuclear cell (PBMC) before and after immunotherapy will be analyzed with ELISPOT technology. The efficacy evaluation also includes ctDNA detection before and after neoantigen immunotherapy.

The observed indicators also include physical examination, vital signs, Eastern Cooperative Oncology Group (ECOG) score, weight, blood routine, urine routine, liver and kidney function, coagulation function and tumor markers. At weeks 8, 12, 24, 36, and 48 of the study periods, patients' target lesions will be scanned using enhanced CT or MRI to evaluate the therapeutic effect. The intervention and evaluation schedule are shown in Table1.

For peer review only - http://bmjopen.bmj.com/site/about/guidelines.xhtml

Table 1 Intervention and assessment schedule for the INP Trial

Execute project	W-4~W0 Screening period	W1	W2	W3	W4	W5	W6	W8	W10	W12	W20	W24	W36	W48
Sequencing /Neoantigen preparation	√													
Neoantigen immunization		√ √	√	√	√					√	√			
Physical examination	√	√	√	√	√	√	√	√	√	√	√	√	√	√
Laboratory examination	√ *				√			√		√	√	√	√	√
CT/MRI	√ *							√		√		√	√	√
ctDNA detection	√ *				√			√		√		√	√	√
IFN-γ, CD4+ T cells, CD8+ T cells	√ *				√						√			√
AEs(CTCAE)	√ *	√	√	√	√	√	√	√	√	√	√	√	√	√
QLQ(EORTC QLQ-C30)	√ *				√			√		√		√		√
Survival follow-up		√	√	√	√	√	√	√	√	√	√	√	√	√

* Need to screen out qualified neoantigens before relevant testing, √√ Twice a week; *CT* computed tomography, *MRI* magnetic resonance imaging, *AE* adverse event, *CTCAE* Common Terminology Criteria for Adverse Events, *QLQ* Quality-of-Life Questionnaire.

For peer review only - http://bmjopen.bmj.com/site/about/guidelines.xhtml

Sample size

To the best of our knowledge, there are no objective data on the efficacy of individualized neoantigens in advanced tumors. This study will not stipulate a sample size a priori. The sample size is not therefore determined by power calculations. The purpose of the trial is to evaluate the safety of individualized neoantigens, similar to the phase I clinical trials of drugs, so our sample size is determined to be 20 patients.

Statistical considerations

First, we need to list the number of selected and completed cases, confirm three data sets: full analysis set (FAS), per-protocol set (PPS), safety data set (SS), and then describe demographic data and other baseline characteristic values.

Descriptive data analysis will be used to evaluate the experimental data. The percentage of subjects with medication compliance greater than 80%, and the percentage of subjects with combination medication will be calculated. AEs will be coded according to ICH Dictionary of International Medical Terms (MedDRA). List AE, SAE, major AE, AE leading to disengagement, and calculate the incidence. Data from patients who received at least 5 sessions of individualized tumor neoantigen polypeptide immunotherapy will be included in the clinical outcome and safety assessment. GraphPad Prism 8 will be used to plot the ctDNA dynamic

For peer review only - http://bmjopen.bmj.com/site/about/guidelines.xhtml

curves and immune response curves.

Patient and Public Involvement

Patients and the public will understand the design and benefits of our trial through consulting with patient advisors and reviewing recruitment information. The patients do not participate in the design of the experiment. The results of the experiment will be kept confidential to the patients.

Ethics

The procedures set out in this trial protocol are designed to ensure that all persons involved in the trial abide by the International Conference on Harmonization (ICH) harmonized tripartite guideline on Good Clinical Practice (ICH-GCP) and the ethical principles described in the applicable version of the Declaration of Helsinki. The trial will be carried out in keeping with local legal and regulatory requirements. The regulations of the GCP regulations will be respected. Before the start of the trial, all documents have been submitted to the independent ethics committee. The study protocol has been approved by local ethics committee on Nov 21, 2019 (Protocol version 1.2 (27.09.2019)).

Data quality assurance

The histologic diagnosis will be verified by one of the experienced

For peer review only - http://bmjopen.bmj.com/site/about/guidelines.xhtml

pathologists. Tumor grading and evaluation of post-treatment curative effect should be decided by public consultation with experienced clinicians and radiologists.

To ensure the quality of the data, we will follow the standard operating procedures (SOP) of the Clinical Trial Quality Management Practice Guidelines (CPMP/ICH/135/95) throughout the trial. During the clinical trial process, the supervisor will send the case report form (CRF) to the data management unit in real time, and two data administrators will perform independent double entry and double check. Inconsistent results will be checked and corrected item by item according to CRF.

Trial status

The trial started in September 2019 and is currently recruiting. The length of clinical phase is about 60 months. (The planned end of the study is the end of 2024). The first patient was treated in August 2020.

Discussion

Tumor neoantigens are derived from somatic cell mutations, which are present in various tumors, and there is a strong heterogeneity among them[32]. Cancer neoantigen therapy was first reported in 1979, and this study showed that successfully treated lymphoma patients responded less well to tumor neoantigen therapy[33]. Further studies also suggest that patients with

For peer review only - http://bmjopen.bmj.com/site/about/guidelines.xhtml

low mutation burden may be limited in their use of neoantigen therapy due to the restricted number of immunogenic epitopes[34]. In view of this, our study included patients with a high mutation load.

Current studies suggest that T-cell-based immune responses are involved in the process of tumor neoantigen immunotherapy[10 28 35 36]. The most striking feature of T cells is their memorability, which reflects the long-term and sustained clinical results achieved by tumor immunotherapy. Wedén S et al followed up for 10 years in patients with pancreatic cancer and found that survival was 20% (4/20) in patients treated with a k-RAS mutant neoantigen vaccine compared to 0% (0/87) in the control group[37]. The study by Schreiber's group highlights the critical role of MHC-II in the presentation of neoantigens. MHC-II can induce the polarization of Th1, promote the activation of Cytotoxic T lymphocytes (CTL), and increase the anti-tumor ability of CTL[38 39].

The personalized neoantigen vaccine based on high-throughput sequencing has the advantages of accurately identifying and killing tumor cells, which is expected to be an important breakthrough for vaccines towards clinical practice. As a specific target of immunotherapy, the anti-tumor effect of tumor neoantigens can only target tumor cells rather than normal tissues[40]. The identification of neoantigens and the development of neoantigens vaccine may provide more options for clinical immunotherapy. It may become one of the effective ways to treat malignant tumors in the

For peer review only - http://bmjopen.bmj.com/site/about/guidelines.xhtml

future. This trial will be able to evaluate the safety and efficacy of neoantigen therapy in genomic unstable advanced solid malignant cancer patients and reveal the potential immune effects of individualized neoantigen tumor peptides mixtures in cancer patients.

Ethics and dissemination

Research ethics approval

This study has received approval from the Ethics Committee of Chongqing University Cancer Hospital on Nov. 21, 2019 (207/2019). The study protocol, informed consent form and other submitted documents were reviewed and approved. The trial registration number is ChiCTR1900025364 (http://www.chictr.org.cn/index.aspx).

Confidentiality

During the clinical trial, subjects will be identified solely by means of their individual identification code. Trial data will be stored and encrypted in accordance with local data protection laws.

Dissemination policy

Regardless of the results of the research, the final data will be publicly released. After the data collection is completed (within 12 months of the end of the study), a report with the results of the research will be submitted in an appropriate open-access, peer-reviewed medical journal.

Acknowledgments

This work was supported by Beijing Neoantigen Biotechnology Co., Ltd.in terms of technology. We acknowledge financial support from Special Project for Performance Incentive and Guidance of Scientific Research Institutions of Chongqing, China grant to LW and XC, and Natural Science Foundation and Science and Technology Bureau joint Health Bureau of Chongqing, China grant to JXT. We also thank the patient advisers for their efforts throughout the study.

Contributors

LW, JXT, XC, WYT, JZ and BL developed and planned this trial, and WQN is the

For peer review only - http://bmjopen.bmj.com/site/about/guidelines.xhtml

principal investigator of the study. LW performed basic research and wrote the manuscript. JXT is responsible for statistical planning and statistical analysis. All authors read and approved the final manuscript.

Funding

The trial is sponsored by Special Project for Performance Incentive and Guidance of Scientific Research Institutions of Chongqing, China (cstc2019jxjl130029), Special Project for Performance Incentive and Guidance of Scientific Research Institutions of Chongqing, China (cstc2018jxjl130057), Science and Technology Bureau joint Health Bureau, Chongqing, China (2021MSXM177) and Natural Science Foundation of Chongqing, China (cstc2019jcyj-msxmX0623).

Competing interests

The authors declare that they have no competing interests.

Ethics approval

Ethics Committee of Chongqing University Cancer Hospital.

Provenance and peer review

Not commissioned, externally peer reviewed.

REFERENCES

1. Heron M. Deaths: Leading Causes for 2017. Natl Vital Stat Rep. 2019. 68(6): 1-77.
2. Wang M, 0000-0001-9748-5486 AO, Munoz J, *et al.* KTE-X19 CAR T-Cell Therapy in Relapsed or Refractory Mantle-Cell Lymphoma. *N Engl J Med* 2020;382:1331-42.
3. Liu E, Marin D, Banerjee P, *et al.* Use of CAR-Transduced Natural Killer Cells in CD19-Positive Lymphoid Tumors. *N Engl J Med* 2020;382:545-53.
4. Sagnella SM, Yang L, Stubbs GE, *et al.* Cyto-Immuno-Therapy for Cancer: A Pathway Elicited by Tumor-Targeted, Cytotoxic Drug-Packaged Bacterially Derived Nanocells. *Cancer Cell* 2020;37:354-70.e7.
5. Flatmark K, 0000-0001-7409-0780 AO, Torgunrud A, *et al.* Peptide vaccine targeting mutated GNAS: a potential novel treatment for pseudomyxoma peritonei. *J Immunother Cancer* 2021;9.
6. Hua H, Zhang H, Chen J, *et al.* Targeting Akt in cancer for precision therapy. *J Hematol Oncol* 2021;14:128.
7. Zhou X, Meng Z, She J, *et al.* Near-Infrared Light-Responsive Nitric Oxide Delivery Platform for Enhanced Radioimmunotherapy. *Nanomicro Lett* 2020;12:100.
8. Zhang H, Pandey S, Travers M, *et al.* Targeting CDK9 Reactivates Epigenetically Silenced Genes in Cancer. *Cell* 2018;175:1244-58.e26.

For peer review only - http://bmjopen.bmj.com/site/about/guidelines.xhtml

9. Venook AP, Niedzwiecki D, Lenz HJ, *et al.* Effect of First-Line Chemotherapy Combined With Cetuximab or Bevacizumab on Overall Survival in Patients With KRAS Wild-Type Advanced or Metastatic Colorectal Cancer: A Randomized Clinical Trial. *JAMA* 2017;317:2392-401.

10. Blass E, Ott PA. Advances in the development of personalized neoantigen-based therapeutic cancer vaccines. *Nat Rev Clin Oncol* 2021;18:215-29.

11. Ott PA, Hu Z, Keskin DB*, et al.* An immunogenic personal neoantigen vaccine for patients with melanoma. *Nature* 201707;547:217-21-.

12. Sonntag K, Hashimoto H, Eyrich M*, et al.* Immune monitoring and TCR sequencing of CD4 T cells in a long term responsive patient with metastasized pancreatic ductal carcinoma treated with individualized, neoepitope-derived multipeptide vaccines: a case report. *J Transl Med* 2018;16:23.

13. Tran E, Turcotte S, Gros A*, et al.* Cancer immunotherapy based on mutation-specific CD4+ T cells in a patient with epithelial cancer. *Science* 2014;344:641-5.

14. Liu XS, Mardis ER. Applications of Immunogenomics to Cancer. Cell. 2017. 168(4): 600-612.

15. Tran E, Robbins PF, Rosenberg SA. 'Final common pathway' of human cancer immunotherapy: targeting random somatic mutations. Nat Immunol. 2017. 18(3): 255-262.

16. Vormehr M, Diken M, Boegel S, Kreiter S, Türeci Ö, Sahin U. Mutanome directed cancer immunotherapy. Curr Opin Immunol. 2016. 39: 14-22.

17. Schumacher TN, Schreiber RD. Neoantigens in cancer immunotherapy. Science. 2015. 348(6230): 69-74.

18. Xu H, Zheng X, Zhang S*, et al.* Tumor antigens and immune subtypes guided mRNA vaccine development for kidney renal clear cell carcinoma. *Mol Cancer* 2021;20:159.

19. Yang X, Fan J, Wu Y*, et al.* Synthetic multiepitope neoantigen DNA vaccine for personalized cancer immunotherapy. *Nanomedicine* 2021;37:102443.

20. Quandt J, Schlude C, Bartoschek M*, et al.* Long-peptide vaccination with driver gene mutations in p53 and Kras induces cancer mutation-specific effector as well as regulatory T cell responses. *Oncoimmunology* 2018;7:e1500671-1500671.

21. Carreno BM, Magrini V, Becker-Hapak M*, et al.* Cancer immunotherapy. A dendritic cell vaccine increases the breadth and diversity of melanoma neoantigen-specific T cells. *Science* 2015;348:803-8.

22. Lu YC, Robbins PF. Cancer immunotherapy targeting neoantigens. Semin Immunol. 2016. 28(1): 22-7.

23. Chen X, Yang J, Wang L, Liu B. Personalized neoantigen vaccination with synthetic long peptides: recent advances and future perspectives. Theranostics. 2020. 10(13): 6011-6023.

24. Sahin U, Türeci Ö. Personalized vaccines for cancer immunotherapy. Science. 2018. 359(6382): 1355-1360.

25. Benvenuto M, Focaccetti C, Izzi V*, et al.* Tumor antigens heterogeneity and

For peer review only - http://bmjopen.bmj.com/site/about/guidelines.xhtml

immune response-targeting neoantigens in breast cancer. *Semin Cancer Biol* 2021;72:65-75.

26. Repáraz D, Ruiz M, Llopiz D, *et al.* Neoantigens as potential vaccines in hepatocellular carcinoma. *J Immunother Cancer* 2022;10.

27. Bhojnagarwala PS, Perales-Puchalt A, Cooch N, *et al.* A synDNA vaccine delivering neoAg collections controls heterogenous, multifocal murine lung and ovarian tumors via robust T cell generation. *Mol Ther Oncolytics* 2021;21:278-87.

28. Keskin DB, Anandappa AJ, Sun J, et al. Neoantigen vaccine generates intratumoral T cell responses in phase Ib glioblastoma trial. Nature. 2019. 565(7738): 234-239.

29. Platten M, 0000-0002-4746-887X AO, Bunse L, *et al.* A vaccine targeting mutant IDH1 in newly diagnosed glioma. *Nature* 2021;592:463-8.

30. Cheson BD, Horning SJ, Coiffier B, et al. Report of an international workshop to standardize response criteria for non-Hodgkin's lymphomas. NCI Sponsored International Working Group. J Clin Oncol. 1999. 17(4): 1244.

31. Aaronson NK, Ahmedzai S, Bergman B, et al. The European Organization for Research and Treatment of Cancer QLQ-C30: a quality-of-life instrument for use in international clinical trials in oncology. J Natl Cancer Inst. 1993. 85(5): 365-76.

32. Alexandrov LB, Nik-Zainal S, Wedge DC, *et al.* Signatures of mutational processes in human cancer. *Nature* 2013;500:415-21.

33. King GW, Grozea PC, Eyre HJ, *et al.* Neoantigen response in patients successfully treated for lymphoma. A Southwest Oncology Group study. *Ann Intern Med* 1979;90:892-5.

34. Martin SD, 0000-0001-5914-0305 AO, Brown SD, *et al.* Low Mutation Burden in Ovarian Cancer May Limit the Utility of Neoantigen-Targeted Vaccines. *PLoS One* 2016;11:e0155189.

35. Hu Z, Leet DE, 0000-0002-4494-1705 AO, *et al.* Personal neoantigen vaccines induce persistent memory T cell responses and epitope spreading in patients with melanoma. *Nat Med* 2021;27:515-25.

36. Yamamoto TN, 0000-0003-1965-7822 AO, Kishton RJ, *et al.* Developing neoantigen-targeted T cell-based treatments for solid tumors. *Nat Med* 2019;25:1488-99.

37. Wedén S, Klemp M, Gladhaug IP, et al. Long-term follow-up of patients with resected pancreatic cancer following vaccination against mutant K-ras. Int J Cancer. 2011. 128(5): 1120-8.

38. Alspach E, Lussier DM, Miceli AP, et al. MHC-II neoantigens shape tumour immunity and response to immunotherapy. Nature. 2019. 574(7780): 696-701.

39. Marty PR, Thompson WK, Salem RM, *et al.* Evolutionary Pressure against MHC Class II Binding Cancer Mutations. *Cell* 2018;175:416-28.e13.

40. Yarchoan M, Johnson BA 3rd, Lutz ER, Laheru DA, Jaffee EM. Targeting neoantigens to augment antitumour immunity. Nat Rev Cancer. 2017. 17(4): 209-222.

For peer review only - http://bmjopen.bmj.com/site/about/guidelines.xhtml

Figure Legends

Fig. 1 Design flow chart of neoantigens. *Abbreviations*: *DNA* deoxyribonucleic acid, *RNA* ribonucleic acid, *HLA* human leucocyte antigen, *QC* quality control

Fig.2 The study flow chart

For peer review only

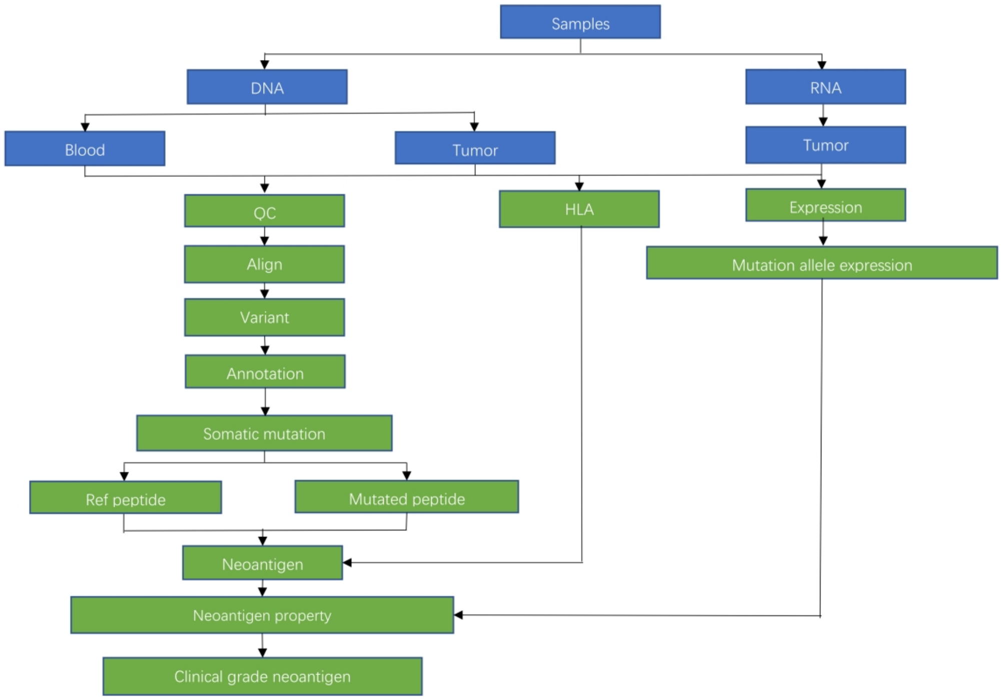

Design flow chart of neoantigens. Abbreviations: DNA deoxyribonucleic acid, RNA ribonucleic acid, HLA human leucocyte antigen, QC quality control

1157x811mm (76 x 76 DPI)

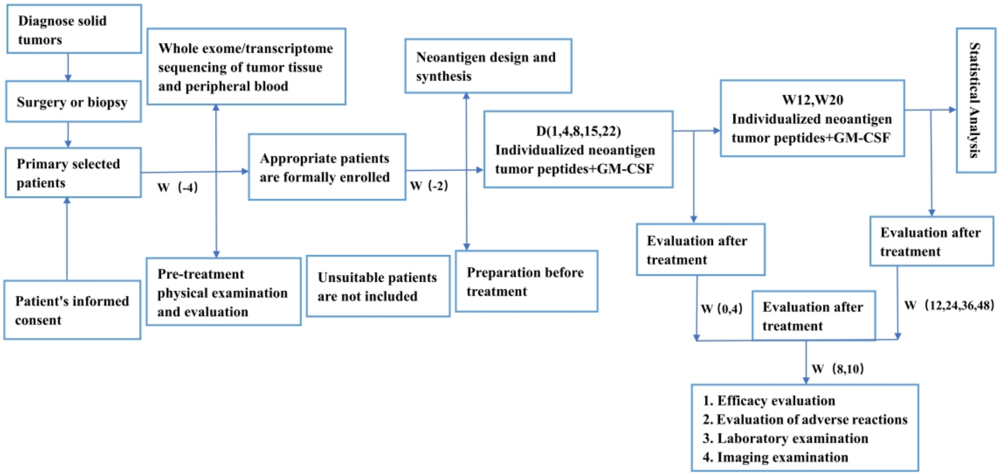

The study flow chart

2214x1050mm (76 x 76 DPI)

Reporting checklist for protocol of a clinical trial.

Based on the SPIRIT guidelines.

Instructions to authors

Complete this checklist by entering the page numbers from your manuscript where readers will find each of the items listed below.

Your article may not currently address all the items on the checklist. Please modify your text to include the missing information. If you are certain that an item does not apply, please write "n/a" and provide a short explanation.

Upload your completed checklist as an extra file when you submit to a journal.

In your methods section, say that you used the SPIRITreporting guidelines, and cite them as:

Chan A-W, Tetzlaff JM, Gøtzsche PC, Altman DG, Mann H, Berlin J, Dickersin K, Hróbjartsson A, Schulz KF, Parulekar WR, Krleža-Jerić K, Laupacis A, Moher D. SPIRIT 2013 Explanation and Elaboration: Guidance for protocols of clinical trials. BMJ. 2013;346:e7586

		Reporting Item	Page Number
**Administrative information**			
Title	#1	Therapy of genomic unstable solid tumors（WHO grade 3/4）in clinical stage III/IV using individualized neoantigen tumor peptides - INP Trial (Individualized neoantigen tumor peptides Immunotherapy):study protocol for an open-label, non-	1

For peer review only

		randomized, prospective, single-arm trial	
Trial registration	#2a	ChiCTR1900025364	3
Trial registration: data set	#2b	N/A	
Protocol version	#3	1.2 (27.09.2019)	15
Funding	#4	The trial is sponsored by Special Project for Performance Incentive and Guidance of Scientific Research Institutions of Chongqing, China (cstc2019jxjl130029), Special Project for Performance Incentive and Guidance of Scientific Research Institutions of Chongqing, China (cstc2018jxjl130057), Science and Technology Bureau joint Health Bureau, Chongqing, China (2021MSXM177) and Natural Science Foundation of Chongqing, China (cstc2019jcyj-msxmX0623).	19
Roles and responsibilities: contributorship	#5a	**Authors:** Ling Wang[1] †, jiaxi Tang[2] †, Xia Chen[3], Juan Zhao[1], Wanyan Tang[1], Bin Liao[1], Weiqi Nian[4,1]*	1
		**Affiliations:**	
		1 Department of Phase I Clinical Trial Ward, Chongqing University Cancer Hospital.	1
		2 Department of Anesthesiology, Chongqing University Cancer	

For peer review only

		Hospital.<br><br>3 Department of Clinical Trial Center, Chongqing University Cancer Hospital.<br><br>4 Department of Oncology, Chongqing Hospital of Traditional Chinese Medicine.	
		**roles of protocol contributors：**<br><br>LW, JXT, XC, WYT, JZ and BL developed and planned this trial, and WQN is the principal investigator of the study. LW performed basic research and wrote the manuscript. JXT is responsible for statistical planning and statistical analysis. All authors read and approved the final manuscript.	19
Roles and responsibilities: sponsor contact information	#5b	Weiqi Nian， Department of Oncology, Chongqing Hospital of Traditional Chinese Medicine, Chongqing, 400013,China. nwqone@gmail.com	1
Roles and responsibilities: sponsor and funder	#5c	Research sponsors and funders participate in the role of research WQN designed the trial. LW performed basic research and wrote the manuscript. JXT is responsible for statistical planning and statistical analysis. XC is responsible for planning and developing the trial, and they jointly determine the decision to submit the report for publication, and they have the final decision on these activities,	19<br><br>N/A
Roles and responsibilities: committees	#5d	LW, JXT, XC, WYT, JZ and BL developed and planned this trial, and WQN is the principal investigator of the study. LW performed basic research and wrote the manuscript. JXT is responsible for statistical planning and statistical analysis. All authors read and approved the final manuscript.	19

For peer review only

**Introduction**			
Background and rationale	#6a	manuscript pages 3-5	3-5
Background and rationale: choice of comparators	#6b	N/A	
Objectives	#7	The trial aims to evaluate the safety and efficacy of individualized tumor neoantigen peptides mixtures in the treatment of genomic unstable advanced solid malignant tumors.	6
Trial design	#8	This is an open-label, non- randomized, prospective, single-arm trial	5
**Methods: Participants, interventions, and outcomes**			
Study setting	#9	Phase I Trial Ward of Tertiary Class A Cancer Hospital	6
Eligibility criteria	#10	Inclusion criteria<br>1. Gender is not limited<br>2. Patients range in age from 18 to 70 years old<br>3. Patients who have progressed after comprehensive treatment and diagnosed with grade 3/4 (World Health Organization	8,9

For peer review only

		(WHO)) genomic unstable (tumor mutational burden (TMB) >10 or microsatellite instability-high (MSI-H)) solid malignant tumors, and lack further effective treatment, or the guidelines at the time of diagnosis of the patient have no corresponding recommended treatment plan are eligible<br>4. There is at least one lesion that can be evaluated.	
			9
		**Exclusion criteria**<br>1. Patients who have a history of hypersensitivity to the testing drug ingredients<br>2. Patients with a previous history of gene therapy or any history of anti-tumor therapy in the last 4 weeks<br>3. Female patients who are currently pregnant, suspected of being pregnant, or expect to become pregnant during the study period<br>4. Patients who are judged to be inappropriate as study subjects.<br>5. Patients who lack sufficient tumor tissues and peripheral blood samples for gene sequencing	
Interventions: description	#11a	Individualized tumor peptide mixtures of 20 antigenic peptides will be injected subcutaneously for therapeutic intervention in	10-11

For peer review only

patients. The benchmark dose is 0.3mg for each antigen peptide, and the single injection dose is 0.3mg×20=6.0mg. GM-CSF will be selected as a polypeptide adjuvant, with the dosage of 0.5 mcg/kg/dose.

The medicine combine individualized tumor antigen polypeptides and GM-CSF will be injected subcutaneously at the lower edge of the upper arm deltoid muscle. The immunotherapy injection cycle is divided into two phases: initial immunization injection and booster immunization injection, which are completed in days 1, 4, 8, 15, 22, and weeks 12, 20. During the initial injection of individualized neoantigen tumor peptides, AEs will be recorded on the day of injection. During booster immunization, AEs will be recorded for 3 consecutive days after each injection. The efficacy of immunotherapy will be evaluated at 4, 8, 12, 24, 36, and 48 weeks. The timeline is displayed in Fig. 2.

The individual follow-up for each participant is 12 months. Patients may enter in a succeeding phase (extended follow-up) according to the preset standards. The trial started in Q3/2019 and will last about 5 years.

Interventions: modifications	#11b	Patients can request withdrawal from the clinical trial at any time. If it becomes difficult to administrate the testing drug to the subject due to the following reasons, the responsible physician or the sharing physician will decide to discontinue the drug administration.	9,10

For peer review only

1. Any time it is found that the patient does not meet the inclusion criteria or meets the exclusion criteria
2. Uncontrollable T cell proliferation
3. SAEs, regardless of whether they are related to treatment
4. Tumor progression.

Interventions: adherance	#11c	According to the Clinical Trial Quality Management Practice Guidelines (CPMP/ICH/135/95), we need to follow the written operating standard procedures (SOP). Quality control applies to every stage of data processing. Take the following steps to ensure the accuracy, consistency, completeness and reliability of the data：Researcher meeting, Research center review of main efficacy variables, Routine research center monitoring, Continuous research center communication and training, Data management quality control check Continuous data acquisition and cleanup, Internal review of data, Quality control inspection of final clinical study report, Partner performs all monitoring duties. The inspectors from Beijing New Kangyuan Biotechnology Co. Ltd. will carry out their work in accordance with the SOP of Beijing New Kangyuan Biotechnology Co.Ltd. The inspector will establish and maintain regular contacts between the investigator, partner and sponsor. They will arrange the supply of test drugs to ensure that the storage conditions of the test drugs meet the requirements. The investigator should verify laboratory test results that are outside the clinically acceptable range, both before and after treatment. If they are caused by diseases other	N/A

For peer review only

		than experimental diseases or diseases not permitted to be combined, they should be reexamined in time. If they are truly abnormal, they should not be included before treatment and should be followed up to normal after treatment.	
Interventions: concomitant care	#11d	Related additional diseases that occurred at the time of informed consent are regarded as concomitant diseases and will be recorded on the corresponding page of the case report form (CRF). The first abnormality or deterioration (intensity, frequency) in the trial is an adverse event (AEs) and must be recorded on the corresponding page of the CRF. If there is no effect on the outcome measurement used in this study and does not interfere with the trial drug, treatment of concomitant diseases that are not restricted by the exclusion criteria is allowed. If drugs are used at the same time, these must be recorded in the patient file and CRF. Adverse reactions/effects after injection of antigen peptides and adjuvants, such as fever, rash, laryngospasm, dyspnea, shock, can be treated with corresponding drugs (bupleurum, fenbid, ibuprofen, aspirin, antihistamines, Anti-allergic and anti-shock drugs). After the injection of antigen peptides and adjuvants, patients with corresponding clinical symptoms (such as systemic infection) due to hypogammaglobulinemia can be treated with gamma globulin. After the injection of antigen peptides and adjuvants, if acute inflammatory response syndrome related to cytokine storm occurs, give a dose of tocilizumab, which can be repeated if necessary. If life-threatening acute inflammatory response	N/A

For peer review only

syndrome occurs, large Dosage hormones. Subjects can receive fully supportive treatment according to the treatment standards as needed.

If no serious side effects occur, adrenal glucocorticoids are not allowed. During the research and treatment process, the following treatments are not allowed: systemic anti-cancer treatment (chemotherapy, immunotherapy, stem cell transplantation treatment), no other anti-tumor drug treatment within 24 weeks after injection of antigen peptides and adjuvants, and no stem cell transplantation treatment. During the study period, subjects and sexual partners were forced to adopt appropriate contraceptive measures (contraceptives, condoms, intrauterine contraceptive rings, surgical methods or abstinence). Breastfeeding is prohibited during the treatment period and 6 months after treatment.

Outcomes	#12	The primary endpoint is to observe and record all AEs and the incidence of serious adverse events (SAE) (grade 3 or above AE) associated with the study drug within 24 weeks after the administration of the individualized neoantigen tumor polypeptide (Refer to National Cancer Institute Common Terminology Criteria for Adverse Events (NCI CTCAE) version 4.03). After that, the	6-7

For peer review only

subjects will continue to be followed at the preset follow-up points for safety. Secondary endpoints are to evaluate the progression-free survival (PFS), objective response rate (ORR), objective duration of remission (DOR), 1-year survival rate, and overall survival (OS)(based on Enhanced computed tomography/magnetic resonance imaging (CT/MRI)). The efficiency of neoantigen peptides in inducing specific anti-tumor immunity is assessed from peripheral blood samples (based on IFN-γ, CD4+ T cells, CD8+ T cells determined by flow cytometry and enzyme-linked immuneospot (ELISPOT) technique. In addition, the efficacy evaluation also includes the detection of circulating tumor DNA (ctDNA) and the quality of life of cancer patients.

Participant timeline #13

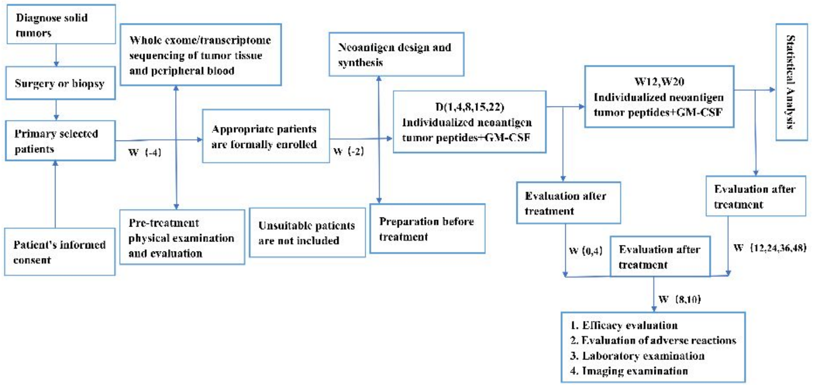

11

For peer review only

Sample size	#14	To the best of our knowledge, there are no objective data on the efficacy of individualized neoantigens in advanced tumors. This study will not stipulate a sample size a priori. The sample size is not therefore determined by power calculations. The purpose of the trial is to evaluate the safety of individualized neoantigens, similar to the phase I clinical trials of drugs, so our sample size is determined to be 20 patients.	14
Recruitment	#15	The hospital where the research site is located is a tertiary-A tumor specialist hospital with sufficient disease sources. In addition, the research hospital has joined a tumor alliance of 20 hospitals, which can also provide adequate and suitable disease sources.	N/A
**Methods: Assignment of interventions (for controlled trials)**		N/A	N/A
Allocation: sequence generation	#16a	n.a., no randomization	N/A
Allocation concealment	#16b	n.a., no randomization	N/A

For peer review only

mechanism			
Allocation: implementation	#16c	n.a., no randomization	N/A
Blinding (masking)	#17a	n.a., no blinded treatment	N/A
Blinding (masking): emergency unblinding	#17b	n.a., no blinded treatment	N/A
**Methods: Data collection, management, and analysis**			
Data collection plan	#18a	To ensure the quality of the data, we will follow the standard operating procedures (SOP) of the Clinical Trial Quality Management Practice Guidelines (CPMP/ICH/135/95) throughout the trial. During the clinical trial process, the supervisor will send the case report form (CRF) to the data management unit in real time, and two data administrators will perform independent double entry and double check. Inconsistent results will be checked and corrected item by item according to CRF.	16
Data collection plan: retention	#18b	Researchers will do their best to ensure that most subjects receive at least one tumor assessment during the treatment	N/A

For peer review only

		cycle. For the main analysis, subjects in the PPS population who withdrew from treatment due to toxicity, early disease progression, or death, in this case, the subjects will be classified as non-responders.	
Data management	#19	A detailed list will be provided in the Investigator Site File. Regardless, there must be a minimum documentation, which provides information on study participation and includes all medical information necessary for appropriate medical care outside of the clinical trial in the patient record. The investigator is responsible for ensuring that all sections of the CRF are completed correctly and that entries can be verified against source data. Any errors should have a single line drawn through them so that the original entry remains legible and the correct data should be entered at the side with the investigator's signature, date and reason for change. Self-explanatory corrections need not to be justified. The correctness of entries in CRF will be confirmed by dated signature of the responsible investigator. During the clinical trial process, the supervisor should send the case report form (CRF) to the data management unit in real time, and the data administrator should perform independent double entry and double check. The original CRF will be transferred to the data management Institute of Medical Biometry and Informatics (IMBI) Chongqing; one copy will be remained by the investigator.	N/A
Statistics:	#20a	First, we need to list the number of selected and completed	14,15

For peer review only

outcomes		cases, confirm three data sets: full analysis set (FAS), per-protocol set (PPS), safety data set (SS), and then describe demographic data and other baseline characteristic values. Descriptive data analysis will be used to evaluate the experimental data. The percentage of subjects with medication compliance greater than 80%, and the percentage of subjects with combination medication will be calculated. AEs will be coded according to ICH Dictionary of International Medical Terms (MedDRA). List AE, SAE, major AE, AE leading to disengagement, and calculate the incidence. Data from patients who received at least 5 sessions of individualized tumor neoantigen polypeptide immunotherapy will be included in the clinical outcome and safety assessment. GraphPad Prism 8 will be used to plot the ctDNA dynamic curves and immune response curves.	
Statistics: additional analyses	#20b	N/A	N/A
Statistics: analysis population and missing data	#20c	N/A	N/A

Methods: Monitoring

Data monitoring:	#21a	The data monitoring committee (DMC) is a group of experts with	N/A

For peer review only

formal committee		various professional knowledge and experience, independent of the sponsor and the organizations that have interests with the sponsor. The DMC is established to independently review and evaluate accumulating safety data from an ongoing clinical trial in order to protect the safety of the subjects and to evaluate the evolving risk-benefit if required.	
Data monitoring: interim analysis	#21b	The trial can be stopped by the LKP if the DMC detects an unexpected accumulation of side effects (each grade 5, 2 succeeding grade 4 or 3 succeeding grade 3 CTC side effects), has new information about the effect-risk ratio of the investigated therapy or the used methods of examination. All investigators have to be informed immediately about a stopping or a permanent ending of the trial. The participating sites must accept the decision. A participating site can also be closed early by the LKP if the site does not act according to ICH-GCP or according to the trial protocol or the recruitment or the quality of the data is below the expectations. If the DMC suggests an interruption or an ending of the trial due to SAE evaluations or other reasons, the ethical board and the authorities (BOB and sate authority) have to be informed. The Ethical Board and the authorities can also decide about an interruption or an ending of the trial.	N/A
Harms	#22	By frequent communications (letters, telephone, fax, e-mail), the site monitor will ensure that the trial is conducted according to the protocol and regulatory requirements. Frequency and details of monitoring will be defined in the monitoring manual. If there	N/A

For peer review only

		are major findings during monitoring or an audit, the investigational site might be closed by the LKP.	
Auditing	#23	The sponsor appoints knowledgeable persons as the clinical trial monitors to monitor and report on the progress of the trial and to verify relevant data. The monitors will conduct an initiation visit before initiation, a follow-up visit after the first patient is enrolled, and subsequent routine monitoring every 8-12 weeks.	N/A
**Ethics and dissemination**			
Research ethics approval	#24	This study has received approval from the Ethics Committee of Chongqing University Cancer Hospital on Nov. 21, 2019 (207/2019). The study protocol, informed consent form and other submitted documents were reviewed and approved. The trial registration number is ChiCTR1900025364 (http://www.chictr.org.cn/index.aspx).	18
Protocol amendments	#25	The Coordinating Investigator and all investigators will be given an up-to-date investigator's brochure containing full details of the status of the pre-clinical and clinical knowledge of the study medication. As soon as new information is obtained, an updated version will be supplied or an amendment added to the existing investigator's brochure. The investigator ensures that all team members are informed adequately about the protocol, all amendments to the protocol, the study procedures und study specific duties and tasks. The investigator will maintain a list to delegate tasks to the team members.	N/A
Consent or	#26a	Phase I ward trial physician	N/A

For peer review only

assent			
Consent or assent: ancillary studies	#26b	N/A	N/A
Confidentiality	#27	During the clinical trial, subjects will be identified solely by means of their individual identification code. Trial data will be stored and encrypted in accordance with local data protection laws.	18
Declaration of interests	#28	The authors declare that they have no competing interests.	19
Data access	#29	According to ICH-GCP the investigator(s)/institution(s) must provide direct access to source data/documents for trial related monitoring, audits and regulatory inspection. Each subject has consented - via written informed consent - to direct access to his/her original medical records for trialrelated monitoring, audit and regulatory inspection. Any data to be recorded directly on the CRFs (i.e., no prior written or electronic record of data), and to be considered to be source data must be clearly identified. In the absence of either an audit-trail or limited access for the monitor the electronic record of data must be printed out (certified copy). In case of changes a new print-out has to be made. Regulatory authorities and/ or auditors authorised by the sponsor may request access to all source documents, CRFs, and other trial documentation. The investigator who must provide support at all times for these activities must guarantee direct	N/A

For peer review only

		access to these documents. The investigator will inform the sponsor immediately about a planned inspection	
Ancillary and post trial care	#30	N/A	N/A
Dissemination policy: trial results	#31a	N/A	N/A
Dissemination policy: authorship	#31b	Publication will be prepared under the lead of the principal investigator of the study. The first and last authorship are reserved for the principal investigator and the study coordinator of the study if both do not wish to transfer their authorship to a third person. All data will be published independently of the results of the trial.	N/A
Dissemination policy: reproducible research	#31c	N/A	N/A
**Appendices**			
Informed consent materials	#32	Before being admitted to the clinical trial, the patient must consent to participate after being fully informed by the investigator or a designated member of the investigating team about the nature, importance, risks and individual consequences of the clinical trial and their right, to terminate the participation at any time. The patient should also have the opportunity to consult	N/A

For peer review only

the investigator, or a physician member of the investigating team about the details of the clinical trial. The informed consent to participate in the clinical trial may be withdrawn by the patient verbally in the presence of, or in written form directed to, the investigator or a physician member of the investigating team at any time during the trial. The patient must not entail any disadvantage therefore or be coerced or unduly influenced to continue to participate. Furthermore, the patient is not obligated to disclose reasons for the withdrawal of the consent. If the patient has a primary physician, the investigator should inform him or her about the patient's participation in the trial, provided the patient agrees hereto. After reading the informed consent document, the patient must give consent in writing. The patient's consent must be confirmed by the personally dated signature of the patient and by the personally dated signature of the physician conducting the informed consent discussion. If the patient is unable to write, oral presentation and explanation of the content of the informed consent form and of the data protection information must take place in the presence of an impartial witness. The witness and the physician conducting the informed consent discussions must also sign and personally date the consent document. The witness must not be in any way dependent on the sponsor of the trial, the trial site or any member of the investigating team (e. g. an employee at the trial site.). A copy of the signed informed consent document must be given to the subject; the original will be filed by the investigator.

For peer review only

		The documents must be in a language understandable to the subject and must specify who informed the subject. The subjects will be informed as soon as possible if new information may influence his/her decision to participate in the trial. The communication of this information should be documented. The informed consent for genetic profiling and the extended follow-up according to a patient register will be independent of the consent of participation in the trial.	
Biological specimens	#33	Whole exome and transcriptome sequencing will be tested on the patient's biopsy or surgically removed tumor tissue, and whole exome sequencing will also be performed on the patient's blood sample. This gene sequencing can obtain comprehensive genomic information such as the mutation status of all genes, human leukocyte antigen (HLA) genotypes, and expression levels of mutated genes in the patients, which can be used to confirm patient's entry criteria and synthesize neoantigen tumor peptides. In addition, 2ml peripheral blood samples will be collected and IFN-γ, CD4+, CD8+ antigen signals in peripheral blood will be detected by flow cytometry to evaluate the effectiveness of tumor neoantigen polypeptide in inducing specific anti-tumor immunity. The changes of IFN-γ cells, CD4+T cells, and CD8+T cells in the peripheral blood mononuclear cell (PBMC) before and after immunotherapy will be analyzed with ELISPOT technology. The efficacy evaluation also includes ctDNA detection before and after neoantigen immunotherapy. The observed indicators also include physical examination, vital	7,11,12

For peer review only

signs, Eastern Cooperative Oncology Group (ECOG) score, weight, blood routine, urine routine, liver and kidney function, coagulation function and tumor markers.

None The SPIRIT Explanation and Elaboration paper is distributed under the terms of the Creative Commons Attribution License CC-BY-NC. This checklist can be completed online using https://www.goodreports.org/, a tool made by the EQUATOR Network in collaboration with Penelope.ai

For peer review only
